# Telephone Cognitive-Behavioral Therapy for Subthreshold Depression and Presenteeism in Workplace: A Randomized Controlled Trial

**DOI:** 10.1371/journal.pone.0035330

**Published:** 2012-04-19

**Authors:** Toshi A. Furukawa, Masaru Horikoshi, Norito Kawakami, Masayo Kadota, Megumi Sasaki, Yuki Sekiya, Hiroki Hosogoshi, Masami Kashimura, Kenichi Asano, Hitomi Terashima, Kazunori Iwasa, Minoru Nagasaku, Louis C. Grothaus, on behalf of the GENKI Project

**Affiliations:** 1 Department of Psychiatry and Cognitive-Behavioral Medicine, Nagoya City University Graduate School of Medical Sciences, Nagoya, Japan; 2 National Center of Neurology and Psychiatry, Kodaira, Japan; 3 Department of Mental Health, University of Tokyo Graduate School of Medicine, Tokyo, Japan; 4 Center for Education and Research on the Science of Preventive Education, Naruto University of Education, Naruto, Japan; 5 University of Tsukuba Graduate School of Comprehensive Human Science, Tsukuba, Japan; 6 Faculty of Clinical Psychology, Kyoto Bunkyo University, Kyoto, Japan; 7 Institute of Psychology, University of Tsukuba Graduate School of Comprehensive Human Sciences, Tsukuba, Japan; 8 Faculty of Applied Psychology, Tokyo Seitoku University, Tokyo, Japan; 9 Faculty of Education, Shujitsu University, Okayama, Japan; 10 Faculty of Psychology, Surugadai University, Hanno, Japan; 11 Group Health Research Institute, Group Health, Seattle, Washington, United States of America; University Paris Descartes, France

## Abstract

**Background:**

Subthreshold depression is highly prevalent in the general population and causes great loss to society especially in the form of reduced productivity while at work (presenteeism). We developed a highly-structured manualized eight-session cognitive-behavioral program with a focus on subthreshold depression in the workplace and to be administered via telephone by trained psychotherapists (tCBT).

**Methods:**

We conducted a parallel-group, non-blinded randomized controlled trial of tCBT in addition to the pre-existing Employee Assistance Program (EAP) versus EAP alone among workers with subthreshold depression at a large manufacturing company in Japan. The primary outcomes were depression severity as measured with Beck Depression Inventory-II (BDI-II) and presenteeism as measured with World Health Organization Health and Work Productivity Questionnaire (HPQ). In the course of the trial the follow-up period was shortened in order to increase acceptability of the study.

**Results:**

The planned sample size was 108 per arm but the trial was stopped early due to low accrual. Altogether 118 subjects were randomized to tCBT+EAP (n = 58) and to EAP alone (n = 60). The BDI-II scores fell from the mean of 17.3 at baseline to 11.0 in the intervention group and to 15.7 in the control group after 4 months (p<0.001, Effect size = 0.69, 95%CI: 0.32 to 1.05). However, there was no statistically significant decrease in absolute and relative presenteeism (p = 0.44, ES = 0.15, −0.21 to 0.52, and p = 0.50, ES = 0.02, −0.34 to 0.39, respectively).

**Conclusion:**

Remote CBT, including tCBT, may provide easy access to quality-assured effective psychotherapy for people in the work force who present with subthreshold depression. Further studies are needed to evaluate the effectiveness of this approach in longer terms. The study was funded by Sekisui Chemicals Co. Ltd.

**Trial Registration:**

ClinicalTrials.gov NCT00885014

## Introduction

Subthreshold depression, variably defined as depressive state failing to reach the diagnostic threshold for major depression and sometimes also called subsyndromal depression or minor depression, is highly prevalent, clinically relevant and societally important.

The prevalence of subthreshold depression has been estimated at around 5–20% in the community and in the primary care patients [1], [2], [3]. It is invariably associated with significant psychological suffering, functional impairment and decreased quality of life [1], [4], [5], [6]. Subthreshold depression is also a strong risk factor for major depression both in the short [7] and long [8] terms.

Given this high prevalence and significant morbidities, subthreshold depression represents a substantive burden on the society. Its sufferers also report more days absent from work (absenteeism) and less productivity while at work (presenteeism). Taken altogether, the cost of minor depression may be as high as 160 million US dollars per year per 1 million inhabitants, which is only marginally less than 192 million US dollars per year that is due to major depression [9]. Of particular note is the fact that 98% of this societal cost of subthreshold depression is estimated to be due to lost productivity.

Management of subthreshold depression is therefore a matter of clinical and society urgency. However, the effectiveness of the customary medical treatment for this condition, namely pharmacotherapy, has recently been called into serious question. A systematic review of drug treatments for minor depression identified six studies (468 patients) and concluded that there is unlikely to be a clinically important advantage for antidepressants over placebo in individuals with minor depression [10]. An individual patient data meta-analysis of antidepressant trials pooled six studies (five studies of major depression and one of minor depression; 718 patients) and found that patients with Hamilton Depression Rating Scale (HAM-D) score below 23 at baseline, either due to major depression or due to minor depression, are likely to show an effect size of only 0.2 or less on antidepressants in comparison with placebo [11]. A between-group effect size of 0.2, 0.5 and 0.8 are traditionally interpreted as representing small, moderate and large effect [12].

It is well established that psychological interventions are effective in the treatment of major depressive disorders [13]. A systematic review was recently published that focused on psychological treatments of subthreshold depression. It identified seven trials (700 subjects) and the pooled effect size at post-treatment was 0.42 (95%CI: 0.23 to 0.60, p<0.01) [14]. All but one of these interventions utilized cognitive-behavioral therapy (CBT) or its variant and compared the intervention against the care as usual. It has recently been suggested, however, that the effect size of CBT may be overestimated due to poor methodologic quality of included trials [15] and to probable publication bias [16]. Effectiveness of CBT may also be less clear for milder depression, as patients with major depression with HAM-D scores less than 20 at baseline showed only a non-significant small effect size with wide confidence intervals (0.22, 95%CI: −0.05 to 0.49, p = 0.11) when four relevant trials (753 patients) that examined baseline severity as effect moderator were pooled [17].

Management of subthreshold depression is therefore in need of a greater number of high quality studies. The present study focused on subthreshold depression in the workplace, targeting both depression itself and associated decreased productivity, through the novel CBT method of delivery using the telephone. Remote CBT using telecommunication technology appears to be particularly suited to the problem of depression [18], [19] and presenteeism in the workplace [20], as it obviates the need to commute and enables wider access to quality-assured psychotherapy.

## Methods

### Procedures

The study was approved by the Ethics Review Committee of Nagoya City University Graduate School of Medicine, and is registered at ClinicalTrials.gov (Identifier: NCT00885014). The protocol for this trial and supporting CONSORT checklist are available as supporting information: see Protocol S1, Protocol S2 and Checklist S1.

After full explanation of the purpose and procedure of the study, potential participants were given two opportunities to provide written informed consent. At the initial screening, the participants were asked to provide their written informed consent to fill in the screening questionnaire (Health and Work Performance Questionnaire (HPQ) and K6. Cf. below) and to answer the telephone interview when invited for the ultimate purpose of participating in the intervention study. Those scoring 9 or higher on K6 were then invited for the telephone interview by a trained interviewer using the Composite International Diagnostic Interview, mood and substance sections, and also to fill in the Beck Depression Inventory-II (BDI-II). Finally, those without current major depression, bipolar disorder or substance dependence, not receiving any current treatment for a mental health problem, and scoring 10 or higher on BDI-II were asked to provide their final informed consent to participate in the intervention study.

The participants providing their definitive consent were then randomized 1∶1 either to (1) telephone cognitive-behavior therapy (tCBT) or to (2) waiting list by an independent clinical research coordinator (CRC) at the central office. The random sequence was generated independently by a study statistician, stratified for depression severity at baseline (BDI-II score ≤19 or ≥20), presenteeism in the past month (HPQ item5 ≤5 or ≥6) and study site. The random sequence within each stratum was blocked with variable length, unknown to the CRC or to the study principal investigators. The random sequence was managed by a spreadsheet program which reveals the allocation only after a participant is registered by the CRC in order to guarantee allocation concealment.

Those allocated to the tCBT started the telephone sessions immediately, and were asked to fill in the end of treatment questionnaires (HPQ, K6, BDI-II and service satisfaction questionnaire) within one month after completion of the program. Those allocated to the waiting list had to wait for four months, fill in the end of treatment questionnaire and, if they still desired, could receive the tCBT sessions. Those who received these additional tCBT were asked to fill in the follow-up questionnaire after the end of their treatment.

### Participants

Working men and women with subthreshold depression were recruited at 13 factories and offices of a large manufacturing company (19,742 employees) in Japan. Inclusion criteria were:

1. Age 20–57 at study entry
2. Men and women
3. Currently employed full-time (either regular or temporary)
4. Expected to be employed full-time for 6 months after screening
5. K6 scores greater than or equal to 9 at screening
6. BDI2 scores greater than or equal to 10 at screening

The exclusion criteria were:

1. Major depressive episode in the past month, as ascertained by CIDI (We did not exclude dysthymia or major depression in partial remission)
2. Lifetime history of bipolar disorder, as ascertained by CIDI
3. Any substance dependence in the past 12 months, as ascertained by CIDI
4. Any other current mental disorder if it constituted the predominant aspect of the clinical presentation and required treatment not offered in the study
5. Current treatment for a mental health problem from a mental health professional
6. Sick leave for 6 or more days for a physical or mental condition in the past month
7. Expected to be on pregnancy leave, maternity leave or nursing leave within 6 months after screening

### Sample size calculation

A systematic review of psychological treatments, mostly CBT, for subthreshold depression yielded a Cohen's d of 0.42 (95%CI: 0.23 to 0.60) at post-test [14]. In order to detect an effect size of 0.40 or greater at an alpha error rate of 0.05 and a beta error rate of 0.20, the estimated sample size was 98 participants per arm. With the anticipated dropout rate of 10%, the necessary sample size was 108 participants per arm.

No interim analysis was pre-planned as this was a small study and we had anticipated no serious adverse event with our psychotherapeutic intervention.

### Interventions

The telephone CBT is a structured, manualized 8-session program adapted from a previously established manual [20], [21], [22]. The program was piloted and rewritten extensively in order to fit the Japanese working population with subthreshold symptomatology.

The patient manual, to be shared both by the participant and the therapist, contains all the materials to be covered in each session, with ample space for the participant to write in his/her own examples. A separate therapist manual was prepared, which specifies the actual flow of each session, followed by the therapist's session checklist after each session and sample emails to be sent to the participant between sessions. The emails to be sent to the participant a few days before the next appointed telephone session was meant to serve both as a summary of the previous session, prompter for the agreed-upon homework and reminder of the next session. The therapist manual proved to be a valuable resource in the training of new therapists, with explanation of theoretical background of the tCBT program and pragmatic tips in the actual conduct of its sessions. The participant and the therapist also shared a booklet termed “Activity Pocketbook” which bound all the homework worksheets in a small notebook format that the participant could easily carry around with him/her and jot down his/her self-monitoring results, activity results and automatic thoughts on the go.

Each session was designed for completion in 30–45 minutes but actual lengths varied according to participants' and therapists' assessment of need. Sessions were meant to occur at weekly intervals but the scheduling was flexible to accommodate the working population's work schedule, especially in the latter half of the program. Each session began with a brief structured assessment of depressive symptoms with K6, review of the previous session and the homework. The initial session included psychoeducation of the CBT model and provided the rationale of the whole program. Sessions 2–4 focused on increasing pleasant activities through personal experiments [23]. Sessions 5–7 focused on identifying, distancing from and challenging negative automatic thoughts [24]. In Session 8 the participant and the therapist together reviewed the cognitive and behavioral skills covered in the program and created a personal self-care plan for self-monitoring, identification and preparation for high risk situations, and self-management. All sessions included a motivational assessment of each participant's degree of interest and confidence in completing homework assignments in their daily lives.

Telephone counselors were master-level, doctor-level and postdoctoral clinical psychologists, social workers or nurses with at least 1 year of clinical experiences. We initially invited Dr Evette Ludman, who was one of the original developers of the tCBT program [25], for a two-day workshop, before we developed its Japanese version. After completion of the Japanese worksite version, we required that the telephone counselors receive at least 12 hours of didactic lecture followed by role plays, listen to at least 8 audiotaped sessions, have two clients' therapy (hence 16 sessions) supervised by TAF or MH before they could act as therapists in the current study.

In addition to the structured, manualized style of tCBT and the specific training as described above that the counselors had to complete, the quality of the administered CBT telephone sessions was assured by on-going supervision and consultation. All the therapists had to have at least 3 out of their 8 sessions per participant supervised on an on-going basis through auditotaped recordings throughout the study. The CRC was also to check client adherence and notify the therapist and his/her supervisor if (i) more than three weeks had elapsed between sessions or (ii) K6 was 10 or greater for any of the session #2 through #4 and 6 or greater for sessions #5 or afterwards. In addition, whenever any question or need arose, they were able to consult TAF or MH. The counselors' meeting to discuss the progress of each participant's treatment was held every two months.

Participants assigned to tCBT or to the waiting list condition were free to seek assistance through the Employee Assistance Program (EAP) which was run by a contracted private company, and which included stress diagnostics and reduction program on the web, telephone consultation, and email consultation. Furthermore, they were also free to seek professional help outside the company such as professional counselors and medical doctors.

### Outcomes

#### Beck Depression Inventory-II

The Beck Depression Inventory, originally published in 1961 [26], has been the most widely used self-report measure of depression severity. With the advent of the DSM-IV, the time frame and question items have been updated as the 2nd edition of the BDI, and its reliability and validity have been confirmed [27]. The reliability and validity of the Japanese version have been found to be excellent [28]. The cutoff of 9/10 was chosen rather arbitrarily in the present study because the normative cutoff for mild depression is 13/14 [27], [28]. We lowered this threshold because the study focused on subthreshold depression and we had expected milder cases to be enrolled in this trial.

We administered BDI-II at baseline, at 4-month follow-up (end of treatment), and at 8-month follow-up (for those who were on the waiting list and received the tCBT). BDI-II is the primary outcome of this study.

#### K6

K6 is a recently developed very short (6-item) self-report questionnaire to screen for common mental disorders [29]. It is based on modern item response theory methods and consists of questions that are maximally discriminative of respondents in the 90^th^-99^th^ percentile range of the general population distribution because it is known that between 5–10% of the population suffer from mental disorders at any point in time. Only items displaying constant psychometric characteristics across sociodemographic variation are included in the final model. K6 has been found to work as well as and better than some widely used screening questionnaires [30], [31]. The Japanese version has been validated [32].

We administered K6 as the initial screening instrument. The cutoff of 8/9 was selected according to the Japanese calibration study [32]. In addition, we used K6 as a process measure in the course of the tCBT and administered it at the beginning of every session because it has been increasingly used not only as a screening scale but also as a severity measure [33]. Together with the screening, 4-month follow-up and 8-month follow-up data, K6 is one of the secondary outcome measures.

#### Health and Work Performance Questionnaire

The World Health Organization Heath and Work Performance Questionnaire (HPQ) is a self-report instrument designed to estimate the workplace costs of health problems in terms of self-reported sickness absence (absenteeism) and reduced job performance (presenteeism). Validation studies have found documented significant associations (r = 0.61 to 0.87) of HPQ work hours assessments with payroll records [34] and job performance assessments with supervisor ratings (r = 0.52) [35] and other administrative records (area under the curve, 0.58 to 0.72) [36]. The Japanese version of the HPQ has been used in the World Mental Health Survey in Japan [37].

The HPQ measures of presenteeism are based on the following two questions: “On a scale from 0 to 10 where 0 is the worst job performance anyone could have at your job and 10 is the performance of a top worker, how would you rate the usual performance of most workers in a job similar to yours?” and “Using the same 0-to-10 scale, how would you rate your overall job performance on the days you worked during the past 4 weeks?” The absolute presenteeism score is obtained by multiplying by 10 the respondent's response to the second question. The relative presenteeism score is obtained by dividing the first response by the second response, multiplied by 100.

We administered HPQ at screening, at 4-month follow-up (end of treatment) and at 8-month follow-up. Our primary outcomes included the two presenteeism scores as defined by the HPQ as well as the actual hours worked in the past 4 weeks.

#### Composite International Diagnostic Interview

The CIDI is a widely used fully-structured diagnostic interview for assessing mental disorders, to be used with the general population by trained lay interviewers [38]. It has been successively updated to accommodate the DSM-IV. We used the most recent computerized version [36] and administered the sections for mood disorders and alcohol use. The concordance between the CIDI and standardized clinical assessments has been reported [39]. The Japanese version of the CIDI has been used in the World Mental Health Survey in Japan [37].

#### Service Satisfaction

The participants' satisfaction with the program (tCBT+EAP or EAP alone) was measured with a single-item question “How satisfied are you with the stress management welfare program as provided by your company?” rated between 1 = “Very unsatisfied” through 6 = “Very satisfied.” The program here referred to EAP+tCBT in the intervention group and EAP alone in the control group.

We also asked a few additional ad hoc questions to those who received the tCBT, including “Were you satisfied with the telephone counseling?”, “Would you recommend this program to other people?” and “Would you like to do the program again when you feel under stress?”, on a 6-level Likert scale between 1 = “Very unsatisfied” through 6 = “Very satisfied.”

### Statistical analyses

We compared the means in the control and intervention groups at the 4-month follow-up using a t-test, permutation test, and a maximum likelihood mixed-effects model. The permutation test was used to get an exact distribution for the t-statistic based on a Monte-Carlo simulation with 1,000,000 replications. Permutations were done within the four strata defined by baseline BDI and baseline absenteeism. The mixed model analysis, a type of repeated measures analysis, includes all randomized individuals, including those with missing outcome data at month 4 and it accounts for the within-person correlation between baseline and follow-up. In this model we adjusted for baseline covariates including BDI-II, absenteeism, site, age and gender. The maximum likelihood mixed model accounts for missing data provided that the data are MAR (missing-at-random) conditional on the covariates and the baseline values of the outcome. We decided a priori to make the mixed model our primary analysis since it is intention-to-treat and includes all randomized individuals in the analysis.

For the overall satisfaction score, which did not have the baseline measurements, we used regression (rather than mixed models) to compare month 4 treatment means adjusting for the stratification variables, age and gender.

All the analyses were performed with SPSS Version 18.0 [40] except for the permutation analyses which were done using SAS Version 9.2 [41].

### Changes to the protocol

One major change made to the protocol after trial commencement is the shortening of the waiting period. Originally it was planned that the waiting period would be 15 months but the initial low participation rate prompted us to shorten the waiting period to 4 months after five participants had been enrolled, in order to increase acceptability of the study.

We also had to stop recruitment after we entered 118 patients after 18 months of trial commencement. The participation rate turned out to be much lower than we had anticipated. We calculated that this sample size would still allow us to detect an effect size of 0.5 or greater at an alpha error of 0.05 and a beta error of 0.20.

## Results

### Participants

**Figure 1:**
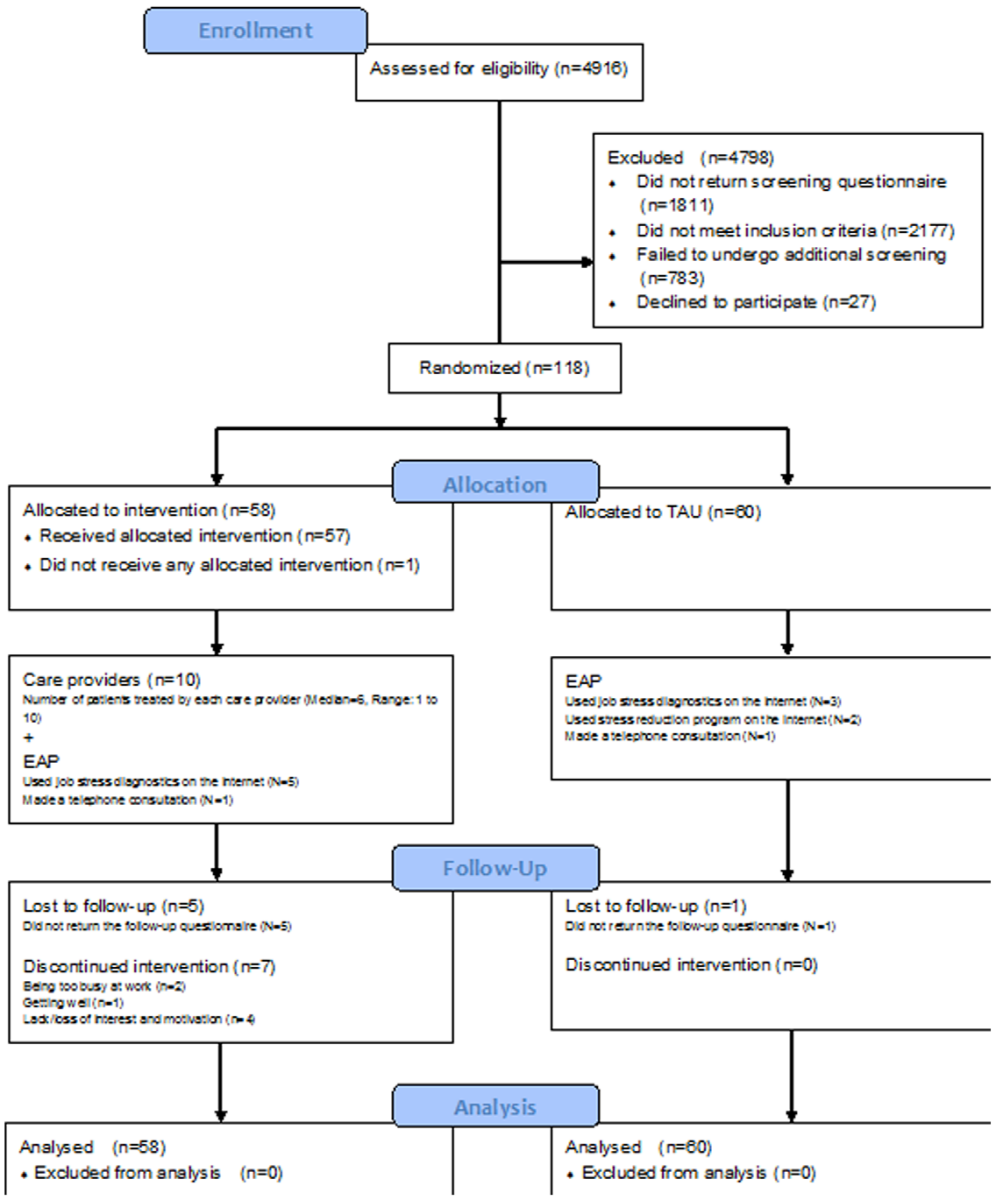
CONSORT participant flow diagram.

Altogether we screened 4916 working men and women at 13 factories and offices between June 2009 and December 2010. 3105 (63.2%) returned the screening questionnaire, of whom 961 scored 9 or higher on K6. Of the latter, 243 were found to satisfy all the eligibility criteria after telephone interviews with CIDI. After the second telephone interview explaining the study purposes and procedures, 118 participants finally gave their written informed consent to be randomized to the intervention or the control arms. There were 10 telephone counselors, each providing tCBT to a median of 6 participants (range: 1 to 10). (Cf. Figure 1 for the CONSORT flow diagram)

Table 1 gives the demographic and clinical variables of the participants. There were no notable differences in these baseline characteristics of the tCBT+EAP and EAP alone groups.

**Table 1:** Demographic and clinical characteristics of the participants.

	EAP+tCBT (n = 58)	EAP (n = 60)
Age, years	39.4 (7.7)	39.3 (8.2)
Males (%)	47 (81%)	45 (75%)
Job category		
Sales/Marketing	20 (35%)	19 (32%)
Production/Factory	10 (17%)	9 (15%)
Engineering/Technical	18 (31%)	15 (25%)
Administration/Management	10 (17%)	16 (27%)
Job rank		
Supervisory	13 (22%)	18 (30%)
Nonsupervisory and other	45 (78%)	40 (70%)
Psychiatric history		
History of major depressive episode	13 (22%)	9 (15%)
History of alcohol dependence	2 (3%)	1 (2%)
BDI-II	17.7 (6.5)	16.8 (5.7)
K6	13.0 (3.0)	12.4 (3.0)
HPQ		
Absolute presenteeism	55.2 (18.9)	56.3 (21.3)
Relative presenteeism	91.3 (33.6)	96.4 (37.7)
Hours worked past 4 weeks	215 (52)	210 (47)

The figures in parentheses are either percentages or SD.

BDI-II: Beck Depression Inventory-II.

HPQ: World Health Organization Health and Work Performance Questionnaire.

### Interventions received and Follow-up questionnaire

Of the 58 participants who were allocated to the intervention arm, 51 participants (87.9%) completed at least 4 or more sessions. Of the seven who dropped out from treatment prematurely, one had no session, two had one session only, one had two sessions only, and further three had only three sessions. The reasons for dropouts as assessed by the therapists included: two for being too busy at work, one for feeling well and feeling no more need, and four for lack/loss of interest and motivation. The mean (SD) and median number of sessions delivered was 7.0 (2.1) and 8 (min = 0, max = 9, IQR:8 to 8), and the average length of a session was 56.1 (12.1) minutes.

Of the seven who prematurely stopped the treatment, three responded to the 4-month follow-up, while the remaining four did not return the questionnaire. Of the 51 who finished at least half the program, only one did not return the follow-up questionnaire. Thus the overall follow-up retention percentage for the whole experimental arm was 91.4%. Of the 60 who were placed on the waiting list, only one participant did not return the 4-month follow-up questionnaire. (Follow-up percentage 98.3%)

During the parallel comparison period, in the EAP group (n = 60), three utilized the job stress diagnostics on the internet page for job-related stress, two used the stress reduction program on the internet page, and one made a telephone consultation. In the EAP+tCBT group (n = 58), five used the job stress diagnostics, and one made a telephone consultation.

After the 4-month follow-up, 36 (60.0%) out of the EAP only group chose to receive the tCBT sessions. 35 of them (97.2%) received at least 4 sessions.

### Effects on depression outcomes

Table 2 shows the group means and their 95% confidence intervals for the BDI-II and K6 scores at the 4-month follow-up, adjusted for the stratification variables, age and sex and taking account of all the randomized participants in a maximum likelihood mixed effects model. The group * times interaction term was highly significant (p<0.001) for both BDI-II and K6. The p-values by the completers' analyses (t-test and permutation test) were virtually identical.

**Table 2:** Comparison of the intervention and control groups at the 4-month follow-up.

	EAP+tCBT (n = 58)	EAP (n = 60)	Effect size	Mixed model p-value	T-test p-value	Permutation test p-value
BDI-II	11.0 (9.2 to 12.8)	15.7 (14.0 to 17.4)	0.69 (0.32 to 1.05)	0.001	0.001	0.0006
K6	6.5 (5.5 to 7.4)	9.0 (8.1 to 9.8)	0.71 (0.34 to 1.07)	<0.001	0.002	0.002
HPQ						
Absolute presenteeism	62.4 (58.1 to 66.7)	59.9 (55.8 to 64.0)	0.15 (−0.21 to 0.52)	0.44	0.56	0.58
Relative presenteeism	104.0 (95.6 to 112.5)	103.3 (95.2 to 111.3)	0.02 (−0.34 to 0.39)	0.50	0.74	0.83
Hours worked past 4 weeks	199 (186 to 212)	190 (178 to 203)	0.18 (−0.18 to 0.54)	0.59	0.37	0.39
Overall satisfaction	4.42 (4.17 to 4.67)	3.57 (3.33 to 3.81)	0.96 (0.57 to 1.35)	<0.001†	<0.001	<0.001

The means and their 95% confidence intervals were estimated from maximum likelihood mixed effects models adjusting for stratification variables, age and gender.

The effect size was calculated from the difference in the means at 4 months divided by their pooled SD.

P-values compare the two groups and are from 1) a mixed model (using all randomized individuals, including those with missing follow-up data) which adjusted for stratification variables, age, and gender, 2) a t-test, and 3) a permutation test done within the 4 strata defined by baseline BDI and absenteeism. Permutation test is based on Monte-Carlo simulation with 1,000,000 samples (used to get exact distribution of t-statistic).

† : Because this variable did not have the baseline measurement, the means at month 4 and their difference were examined by regression models adjusting for stratification variables, age and gender.

BDI-II: Beck Depression Inventory-II.

HPQ: World Health Organization Health and Work Performance Questionnaire.

In terms of the primary outcomes, tCBT showed superiority over its control condition, with an estimated effect size of 0.69 (95%CI: 0.32 to 1.05) for depression severity as measured with BDI-II. The secondary outcomes corroborated the primary results in that the tCBT condition provided benefit in terms of overall psychological distress as measured with K6 (effect size estimate 0.71, 0.34 to 1.07).

### Effects of work performance outcomes

Table 2 also shows the group means and their 95% confidence intervals for the two presenteeism scores and the actual hours worked on the HPQ at 4 months according to the maximum likelihood mixed effects model. The group * time interaction was not statistically significant for any of the work performance measures. Thus the study failed to demonstrate statistically significant effect for presenteeism or for the hours worked in the past month.

### Satisfaction with service

The overall satisfaction score for the services was 3.57 (3.31 to 3.81) for the EAP alone group and 4.42 (4.17 to 4.67) for the tCBT +EAP group (p<0.001).

The evaluation of the tCBT program was satisfactory with at least 85% showing positive responses to the questions “Were you satisfied with the telephone counseling?”, “Would you recommend this program to other people?” and “Would you like to do the program again when you feel under stress?” The positive response rate was 90%, 86% and 85%, respectively.

No serious adverse event, including suicidality, was reported in either the intervention arm or the control arm.

## Discussion

The present study examined the short-term effectiveness of remote CBT using the telephone among working men and women with subthreshold depression up to four months. It reduced depression symptomatology (p<0.001, ES = 0.69, 0.32 to 1.05) and general psychological distress (p<0.001, ES = 0.71, 0.34 to 1.07) but failed to demonstrate significant improvement in productivity (p>0.05, ES ranged between 0.02 to 0.18). The program was acceptable in the workplace as the dropout from active treatment was relatively low at 12.1% and the employees who received the telephone CBT were generally very satisfied.

Our study therefore added further support to the effectiveness of cognitive-behavioral intervention for subthreshold depression [14], and also to the effectiveness of remote CBT [18]. Our results also corroborate the well-established effectiveness of CBT for occupational stress reduction. One meta-analysis has reported an effect size of 0.68 (0.54 to 0.82) [42] and another an effect size of 1.16 (0.46 to 1.87) [43] of CBT interventions in the workplace. Our results, with an effect size of 0.69 (0.32 to 1.05), can be said to be largely in line with these systematic reviews.

The present study however failed to show a statistically significant improvement in presenteeism, as measured by absolute presenteeism, relative presenteeism or total hours worked according to the HPQ. We are aware of two RCTs to date which have demonstrated significant effect on work productivity through enhanced depression treatment including telephone counseling with treatment-seeking workers [20], [44]. The relative increase in productivity, reflecting both absenteeism and presenteeism, was estimated to be 8% [44] and 13% [20]. In our study, all the participants had been fully employed and working and there was no room for improvement in terms of absenteeism.

The strengths of the current study include, first, its explicit screening for subthreshold depression. We have screened the potential participants with two screening questionnaires (K6 and BDI-II) to ascertain their depressive symptomatology and excluded diagnoses of major depression and bipolar disorder through structured interviews (CIDI). Secondly, the quality control of the treatments was rigorous. It followed highly manualized procedures, with a detailed textbook and a workbook to be shared by both the therapist and the participants. With the training and supervision provided for this study, the quality of the therapy was well controlled. Thirdly, the attrition from treatment (10.3%) and missing follow-ups (5.1%) was relatively low in comparison with the previous studies of subthreshold depression or remote CBT which typically ranged around 20–30%. Lastly, we employed the maximum likelihood mixed effects model and accounted for all the randomized individuals, thus adhering to the intention-to-treat principle.

There are, however, several weaknesses to this study. First and foremost, the control condition (waiting list with pre-existing EAP services of the company which, however, turned out to be little utilized) was weak. It can control for the regression towards the mean, the natural course and Hawthorne effect but not the placebo effect or the non-specific general psychotherapeutic effect. The present study has therefore established the value of adding tCBT to the usual care but not the specific effectiveness of cognitive or behavioral interventions delivered in tCBT. Secondly, due to the nature of the intervention in question, we were able to blind neither the participants nor the therapist to the allocated intervention. Moreover, all our primary outcomes (BDI-II and HPQ) depended on patients' self-report and may have led to overestimation of the treatment effect. Third, our study was relatively short term (up to 4 months) and, due to our study design using the wait list condition, was unable to examine the effect of tCBT in reducing depressive symptomatology in the long term or in preventing future major depressive episodes. The most up-to-date systematic review barely failed to find a statistically significant reduction in future depression through CBT with subtheshold depression (RR = 0.70, 0.47 to 1.03, p = 0.07). Such effectiveness of CBT is yet to be ascertained. Fourth, our failure to demonstrate statistically significant effect on presenteeism could be accounted for by our lack of statistical power (as the two studies which demonstrated statistically significant improvement enrolled two- to five-times more patients than ours), limited range of improvement possible among our participants (as ours were all fully employed and working), insensitivity of our outcome measure [35] and/or true inefficacy of our tCBT in addressing presenteeism among working men and women.

In summary, remote CBT, including telephone CBT, may be able to provide easy access to quality-assured psychotherapy for people in the work force who may present with subdiagnostic psychological distress and cannot readily visit health care services on weekdays. From the viewpoint of service providers, it also means efficient use of the scarce resource, that is well trained CBT therapists, as the remote CBT enables delivery of their CBT from anywhere to anywhere. The average length of a session in our study (56.1 minutes) was somewhat longer than we had originally anticipated but are in line with some telephone-administered CBT programs [45], [46]. Its effect size was large and consistent with previous literature. Whether the effectiveness of remote CBT is durable beyond four months and/or it can reduce incidence of depression in the long term, and whether it can boost subdepressed workers' productivity by a small yet important amount are all important themes for future research.

## Supporting Information

Checklist S1 **CONSORT 2010 checklist of information to include when reporting a randomised trial.**
(DOC)

Protocol S1 **The original protocol in Japanese.**
(DOCX)

Protocol S2 **English translation of the original Japanese protocol.**
(DOCX)
