# Supplementary material for: Telephone Cognitive-Behavioral Therapy for Subthreshold Depression and Presenteeism in Workplace: A Randomized Controlled Trial
Source: PLoS One. 2012 Apr 19;7(4):e35330. doi: 10.1371/journal.pone.0035330 (PMC3330821; doi:10.1371/journal.pone.0035330)
Supplement: Protocol S1 — The original protocol in Japanese. (DOCX) [file pone.0035330.s002.docx]

職場における閾値下抑うつおよび労働効率に対する電話認知行動療法：無作為割付比較試験

古川壽亮，堀越勝，川上憲人，佐々木恵，堀越あゆみ，福森崇貴，寺島瞳，永作稔，樫村正美，細越寛樹，丹羽まどか，関屋裕希，浅野憲一，岩佐和典，Louis Grothaus

本臨床試験はclinicaltrials.gov (登録番号: NCT-XXX)に登録されている

# 1. 背景

うつ病は、個人や世界中の社会、とりわけ完全な市場経済の国々において損失が大きい (World Health Organization, 2004). うつ病の治療，低下した生産性，自殺によるコストは、アメリカ合衆国だけで、2000年に830億ドル(約9兆円)にものぼることが推定されている(Greenberg et al., 2003)。この社会的損失は、高血圧についで第2位となっており、糖尿病、心臓疾患、喘息など、他の慢性疾患よりはるかに大きい(Druss et al., 2001)。

また、直接的な治療コストは、うつ病の社会的損失全体の25%しか説明しない。実際は、うつ病の損失の多く(520億ドル、全体の62%)は、職場での労働生産性の低下として生じている(Greenberg et al., 2003)。異なる方法を取っている他の研究者たちも同様の推定に至っている；たとえば、Druss et al. (2001)によれば、うつ病の治療を受けた人の労働コストは、1996年のアメリカ全土で115億ドルとなっている。また別の研究では(Stewart, Ricci, Chee, Hahn, & Morganstein, 2003)、治療の状態を無視してうつ病のすべての労働者をカウントするならば、440億ドルになるとされている。National Comorbidity Survey Replication (Kessler et al, 2006) による推定では、366億ドルとされている。

　さらに注目すべきことは、職場におけるうつ病のコストは、主としてabsenteeism(欠勤)によるものではなく、presenteeism(出勤はしているが労働効率が低下している状態)によるものだということである。たとえば、American Productivity Audit は、大うつ病の労働者におけるうつ病による損失時間を、absenteeismによるものは1.2時間/週、presenteeismによるものは7.2時間/週と推定している(Stewart et al., 2003)。さらに最近の調査では、うつ病は、うつ病の労働者1人あたり、absenteeism 8.7日、presenteeism 18.2日相当の原因となることを明らかにしている(Kessler et al., 2006)。うつ病は本当に、労働者の健康問題の中で、最も損失の大きいものなのである(Wang et al., 2003)。よりホリスティックな見方をすれば、このような試算はすべて、本人の主観的な苦痛や、家族の主観的苦痛を考慮してはいないことを頭にとどめておくことは重要である。

　したがって、いくつかの研究者グループが、職場におけるうつ病の有害な効果を軽減するための方法を探究してきたことは不思議なことではない。労働関連ストレスの軽減を目指した研究論文の中には，数多くの比較試験が報告されており、概して組織的介入、認知-行動論的介入、その他の介入を通じたものとなっているが(Richardson & Rothstein, 2008; van der Klink, Blonk, Schene, & van Dijk, 2001)、労働生産性の低下を検証したものはたった2論文しかない。Rost et al (Rost, Smith, & Dickinson, 2004) は、米国における地域の12プライマリ・ケア施設を受診した労働者について、通常治療よりも強化したケアと、通常治療とを比較した。強化したケアには、プライマリ・ケア医師による通常治療に加えて、ケア・マネージャーによる電話での接触が含まれており、その内容は、うつ症状の再アセスメント、うつ病とその治療についての教育、患者の状態を医師に報告し、治療ガイドラインにそって治療の調整を勧めることであった。2年にわたり、うつ病への介入には限定された効果しか認められなかったが (p=0.09)、生産性は有意に 6.1% (p<0.05)改善し、absenteeism は 有意傾向で22.8% (p=0.06)改善していた。短期的には、その利益は、うつ病の労働者1名あたりで、presenteeism の減少により1491ドル、absenteeismの減少により539ドル 分に相当した。これらの効果は常勤労働者において、より強固であった。

　Wang et al. (2007) は近年、職場における、より積極的なアウトリーチ治療プログラムについて検討している。彼らはまず、様々な地域の16の大企業で労働者をスクリーニングし、その結果、うつ病の可能性があると判断された人が通常治療群または介入群に無作為に割り付けられた。通常治療に割り付けられた人は、スクリーニングの回答内容からうつ病の可能性があることを伝達され、医師に相談するよう勧められた。介入群に割り付けられた人は、系統的な電話介入プログラムを受けた。この介入には、治療ニーズのアセスメント、対面治療の促進、治療アドヒアランスのモニターとサポートが含まれており、対面治療を辞退した人には、構造化された電話精神療法を提供した。介入は抑うつ重症度を有意に減少させただけでなく(p=0.001) 、12ヶ月に渡る有意に効率的な労働時間(p=0.002)と勤務の継続 (p=0.02) を増加させた。この改善は、通常治療に割り当てられた労働者と比べて、週あたり2時間、年あたり2週間に相当した。

　職場における生産性の低下に挑戦する際に見落としてはならないことは、小うつ病、つまり、閾値下抑うつである。その定義からは、完全な大うつ病よりも症状が少ないとされているが、相当な機能障害と関連しており(Judd, Schettler, & Akiskal, 2002; Wells et al., 1989) 、実際のところ、その有病率の高さから、大うつ病よりも2倍の障害日数をもたらすと考えられる(Broadhead, Blazer, George, & Tse, 1990)。さらに、小うつ病はその後の大うつ病発症の最も明確な前兆であると考えられる (Judd, Akiskal, & Paulus, 1997)。

　幸い、精神療法、特に認知-行動論的な精神療法は、閾値下抑うつを改善するだけでなく

(Allart-van Dam, Hosman, Hoogduin, & Schaap, 2007; Spek et al., 2007; Willemse, Smit, Cuijpers, & Tiemens, 2004) 、将来の大うつ病発症を、18ヶ月間は予防する(Clarke et al., 1995; Clarke et al., 2001)という証拠がある。一方で、現存する証拠では、小うつ病に対する薬物療法の効果は、反復性うつ病の再燃予防という文脈を除いて(Geddes et al., 2003)、支持されていない(Dimidjian et al., 2006; Paykel, Freeling, & Hollyman, 1988)。したがって、この無作為比較研究では以下を目的とする：

1. 職場における閾値下抑うつに対するCBTパッケージの効果を示すこと
2. 職場におけるCBTパッケージの経済的価値を示すこと

これらに対応して、本研究における仮説は以下を含む：

1. CBTパッケージは、閾値下抑うつを呈する労働者の抑うつ重症度の減少や、うつ病エピソードの予防において、通常のEAPよりも有意な効果を持つかどうか
2. CBTパッケージは、年間1人あたりの労働パフォーマンスを少なくとも平均100ドル(約10000円)分促進することができるか
   1. これらがすべて労働パフォーマンスの増加(有効労働時間の増加)によるものであるならば、平均時給40ドル (約4000円)の労働者100名のうち、10名はCBTを受け、年間10000ドル分を補うために有効労働時間を月あたり2時間増やさねばならない。
   2. CBTプログラムが退職者0.1名分を予防するならば、企業が労働者の教育その他の目的で支払うコストに加え、年間給与に換算して8000ドル(約80万円)分の利益を上げることになる。

本研究は、TAUをコントロールとした2群並行群間比較試験であり、非盲検化無作為割り付け臨床試験である。

# 2. 目的

## 2.1 主たる臨床的疑問

我々の主たる臨床的疑問は以下のとおりである：“閾値下の抑うつ(subthreshold depression)を呈する企業労働者において、標準的な従業員支援プログラム(employee assistance program: EAP)に、行動活性化(behavioral activation: BA)と認知再構成(cognitive restructuring: CR)からなる電話CBTプログラムを加えることは、EAPのみの場合よりも、プログラム終了後における抑うつの重症度（BDI2により測定）および労働生産性(HPQにより測定)において優れているか？”

したがって、本研究におけるプライマリ・アウトカムは以下のとおりである：

1)無作為割り付けから4ヶ月後の抑うつ重症度(BDI2により測定)

2) 無作為割り付けから4ヶ月後の労働生産性(HPQにより測定)

これら2つのプライマリ・アウトカムは、電話CBTによる介入の効果を判断するために用いられる。

## 2.2. 第2の臨床的疑問

セカンダリ・アウトカムには以下が含まれる：

1. 無作為割り付けから4ヶ月時点での
   1. 抑うつ重症度(K6により測定)
   2. クライエント満足度(会社のメンタルヘルスに関するサポートについての満足度(Visual Analogue Scale (VAS)による)ならびに電話CBTを受けたクライエントに対する詳細な事後アンケート )
2. 無作為割り付けから15ヶ月時点での
   1. 抑うつ重症度(BDI2およびK6により測定)
   2. 労働パフォーマンス
      1. 過去1ヶ月の労働生産性(HPQ)
      2. 仕事の継続：無作為割り付けから15ヶ月時点までに退職したか(はい/いいえ；もし「はい」ならば、いつ退職したのか？これはサバイバル分析(生存分析)を行う際に重要である)
   3. クライエント満足度(VAS)client satisfaction [VAS]
   4. EAPサービスの使用、医療サービスの使用(身体的疾患/精神的疾患)、自殺企図、入院(身体的疾患・精神的疾患)　(無作為割り付けから15ヶ月の間について、はい/いいえ)
   5. 無作為割り付けから15ヶ月の間に大うつ病エピソード、小うつ病エピソードを発症するまでの期間 (～ヶ月目)

## 2.3. サブグループ解析

プログラムの効果は、抑うつの当初の重症度によって異なると考えられる。そこで、閾値下抑うつの高低を層別化要因のひとつとして扱い、これらの事前に規定したサブグループによって解析を行う。治療効果がサブグループ間で有意に異なる(i.e., サブグループ仮説)かどうかを検出するにはサンプルサイズが充分でないため、最終的な解析よりも探索的なもの(仮説を構築するためのもの)となる。治療効果についてはサブグループの交互作用の有無によって検証するが、焦点となるのは記述的なもので、サブグループごとの治療効果のパターンをみることによって行う。解析の数を少なくするために、プライマリ・アウトカム(無作為割り付けから4ヶ月時点のBDI2とHPQ)に焦点を当てる。

## 2.4. 有害事象

本研究の介入から、健康への有害事象は想定されないが、抑うつの重症化(究極的には自殺)の可能性のみが存在する。深刻な希死念慮等が生じた場合を想定し、緊急用の電話番号を事務局に用意する。臨床研究コーディネーター(CRC)は臨床心理士で、緊急電話に最初に対応し、担当の電話カウンセラーおよび臨床スーパーバイザー(古川壽亮、堀越勝、堀越あゆみ)に、適切な対応方法について相談する。何らかの情報源によって得られた、抑うつの重症化、自殺念慮、自殺企図に関する間接的な情報はCRCに伝えられ、CRCは臨床スーパーバイザーに相談し、個々に合わせて臨床的に最も適切な対応がなされる。

抑うつの重症化はプライマリ・アウトカムならびにセカンダリ・アウトカムで測定する。自殺未遂、自殺の完遂および入院については、15ヶ月フォローアップにて測定する。本研究では、ストッピング・ルールを設けない。なぜなら、有害事象が本研究への参加と明らかに関連したものかどうか、また、すべてのサンプルの登録を完了する前に、アウトカム・データが主たる臨床的疑問に答えるに充分なものかどうかを明らかにするだけの充分大きなサンプルを扱わないためである。

研究責任者である古川は、すべての決定について責任を負う。古川は、報告された有害事象と自殺の危険性があるすべてのケースについて、24時間以内に確認する。出張やその他の業務のためにこの役割を果たせないときには、堀越勝、堀越あゆみ、あるいはその他の任命された臨床研究者が参加者の安全を確認する。

# 3. 参加者

## 3.1. 研究母集団

閾値下抑うつを呈する労働者男女が以下の基準にしたがって抽出される：

### 3.1.1. 包含基準

1. エントリー時20～57歳(定年は通常60歳であるため、フォローアップまでの時間を確保するために、60歳よりもかなり前にリクルートする必要がある)
2. 男性および女性
3. 現在フルタイムで雇用されている(正規社員あるいは非正規社員)
4. スクリーニングから2年間はフルタイムで雇用される予定がある
5. スクリーニング時にK6スコアが9点以上
6. 2回目のスクリーニング時(CIDI面接時)のBDI2スコアが10点以上

### 3.1.2. 除外基準

1. パートタイム職員
2. 過去1ヶ月に身体的あるいは精神的な疾患による病欠が6日以上ある
3. スクリーニングから2年以内に、出産休暇・育児休暇・介護休暇を取る予定がある
4. 現在、メンタルヘルスの問題について専門家からなんらかの治療を受けている人
5. 過去1ヶ月間に大うつ病エピソードが存在する(CIDIにより確認：気分変調性障害や、大うつ病の部分寛解は除外しない)
6. 双極性障害の既往がある(CIDIにより確認)
7. 過去12ヶ月間に何らかの物質依存がある(CIDIにより確認：物質乱用は除外しない)
8. その他に現在精神障害があり、それが主たる病像となっていて、本研究で提供されていない治療を要する人

　某企業のすべての候補者(正規社員、非正規社員合計でおよそ1500名)を登録し、候補者には参加者募集の手紙を送付する。介入研究への参加に同意した人と母集団とを比較し、結果の一般化可能性について吟味する。

## 3.2. サンプルサイズの前提と推定

閾値下抑うつに対する心理学的な治療(その多くはCBTであるが)の系統的レビューでは、介入終了後でCohenの dが 0.42 (95%CI: 0.23 - 0.60)、1年後のフォローアップで 0.16 (95%CI: 0.02 - 0.35)という数値を導いている (Cuijpers, Smit, & van Straten, 2007)。Cohenの d が .42というのは、治療効果(アウトカムの測定値における対照群の平均と介入群の平均の差)が、0.42標準偏差分であることを示す。

|  |  |  | 1:1 |  | If 1:2 |  | If 1:3 |  |
| --- | --- | --- | --- | --- | --- | --- | --- | --- |
| alpha | power | Effect size | n in each cell | Total N | n in active treatment | Total N | n in active treatment | Total N |
| 0.05 | 0.90 | 0.4 | **131** | 262 | **99** | 297 | **88** | 350 |
| 0.05 | 0.80 | 0.4 | **98** | 196 |  |  |  |  |

したがって、介入群に99名を、対照群にその2倍(198名)、合計297名を無作為割り付けすると、標準偏差0.40に相当する群間差を想定した治療効果を、90%の検出力で検出することになる。しかしながら、これらの計算は脱落を考慮していない。全体の90%が4ヶ月フォローアップで回答すると考えると、4ヶ月後には介入群が89名、対照群が178名となる。この場合は、90%の検出力で0.42SDに相当するエフェクト・サイズを同定することにあり。本臨床試験における4ヶ月の時点で期待される効果の最良の推定値とまさに等しいものとなる。

15ヶ月後では、4ヶ月に比してかなり小さい効果しか期待されないので(0.16SD相当)、治療効果を同定するだけの充分な検出力はないと考えられる。しかしながら、次回のRCTで予防効果を示すための洞察を得るために、15ヶ月フォローアップを行う。15ヶ月フォローアップは探索的なものである。

　適格性を有する300人の参加者をリクルートできるならば、介入群：対照群を1:2の比でありつける。200名に満たなければ、1:1で割り付ける。

## 3.3. 参加者のリクルート

企業代表者および(または)研究責任者は各事業所を訪れ、可能であれば小グループ単位で被雇用者たちに会う。そして、本研究の目的と手続きの概要を説明し、あらかじめ本プログラムへの関心を高めるようにする。

　CRCは毎月、200名の被雇用者に対して、参加者募集の手紙と質問紙を送付する。このうち、50名が候補者となると考えられ(K6>=9)、CIDI面接者(佐々木、北川、土屋、島田、大江)によってCIDIを受ける。このうち40名が適格になると考えられ、さらにこのうちの30名から最終的な同意が得られると考えられる。この30名が積極的な介入群(n=10)と待機群(n=20)に無作為割り付けされる。9名の電話カウンセラーは、毎月1～2名のクライエントに対して電話CBTを開始する必要がある。この手続きを9ヶ月間あるいは電話CBT群が100名に達するまで繰り返す。待機群の待機期間が長期になることや、割り付けが1:1でないことは、有能な電話カウンセラーの数が限られていることを以て弁明可能であり、説明がなされる。9名のカウンセラーは毎月1～2名のクライエントのセッションを開始する、つまり、どの時点でも3～4名のクライエントを同時に担当する(クライエント1名あたりの電話CBTに2～3ヶ月を要するため)。100名のクライエントをリクルートするためには、9ヶ月間リクルートを続けることが必要となる。待機群は無作為割り付けから15ヶ月以降に電話CBTを受けることができる。

### 3.3.1. 適格性のアセスメント

#### 第1回スクリーニングと事前のIC

参加者募集の手紙、説明書および同意書、スクリーニング質問紙(JCQ, HPQ, K6, フェースシート(氏名、生年月日、性別、電子メールアドレス、電話番号、職種、身体的疾患の治療中か否か、精神的疾患の治療中か否か、現在何らかの理由で休職しているか否か、質問紙の結果についてフィードバックを希望するか否か、CIDI実施の希望日時を問うもの))を、某企業のすべての労働者に送付する。

スクリーニング質問紙への回答とCIDIの実施に同意する労働者は、署名ずみの同意書とスクリーニング質問紙を個別に返送する。

#### 第2回スクリーニング

質問紙が返送され、K6スコアが9点以上だった場合、CIDI面接者は対象者に電話をかけ、CIDIを実施する。本研究におけるCIDIは、気分障害と物質乱用のセクションをカバーしている。

希望者には、CIDI、JCQ、HPQおよびK6の結果に基づいてフィードバックを行う。

#### 最終的なIC

K6が9点以上で、現在の大うつ病、過去12ヶ月の物質依存、および双極性障害の既往の診断基準を満たさない労働者には、CRCがメール(ないし電話)でコンタクトをとり、介入研究への参加に同意するかどうかを尋ねる。

電話にて口頭で同意の意思を示した労働者には、書面での説明書および同意書、BDI2が送付される。

一方。現在大うつ病エピソードにある労働者は、臨床試験からは除外されるが、CRCがコンタクトをとり、産業医ないしは外部の専門家を受診するよう助言する。CRCはそれまでに得られたすべてのデータに基づき、電話CBTよりも専門家に直接受診した方が利益が大きいと判断されたことを手紙により説明する。

### 3.3.2. ベースライン・アセスメント

ベースライン・アセスメントが、介入開始時の真のベースラインを反映するように、第1回目のICとスクリーニング、第2回目のICとBDI2の実施という2段階を設定し、いずれも2週間以内に行う。3週経過しても回答がない場合には、同意が得られなかったものとみなす。

したがって、ベースライン・アセスメントには、以下の変数が含まれる。

#### スクリーニング質問紙から

1. Job Content Questionnaire 🡪 仕事のコントロール、仕事の要求度
2. Health and Productivity Questionnaire 🡪 過去4週間の労働時間、過去4週間の絶対的および相対的absenteeism、過去4週間の絶対的および相対的presenteeism
3. K6
4. 生年月日
5. 性別
6. 職種

7)　身体的疾患の治療中か否か、精神的疾患の治療中か否か

8)　過去1ヶ月の病欠日数

1. スクリーニングから2年以内に、出産休暇・育児休暇・介護休暇を取る予定があるか否か

CIDIおよびその後の質問紙から

1) 気分障害および物質使用障害に関するCIDIの結果

2) 過去2週間についてのBDI2

### 3.3.3. 説明と同意

本研究の目的と手続きについて開示・説明がなされた後に、すべての参加者から書面による同意を得る。候補者には、参加は任意であること、参加後においても理由を告げずに介入およびアセスメントを辞退することができること、本研究への参加・不参加は会社における利益・不利益につながらないことを説明する。

各参加者のデータは、機密性を保つために、順に割り振られた番号で扱われ、インターネットに接続しないコンピュータに保管される。参加者のリストと番号は電子媒体で保管はせず、安全で施錠された場所に紙媒体で保管され、CRCのみがアクセス可能となる。

ICにおける説明では、アドヒアランスを高めるために、スクリーニングのデータから明らかになったクライエントの感受性・脆弱性について、プログラムから得られると考えられる利益について強調する。

しかしながら、本研究ではTAU条件を設けているため、プログラムを売り込み過ぎることもできない。

プログラムの説明のためにパンフレットを用いる。プログラムのタイトルとして、介入の名前を用いることは避けなければならない：そうでなければ、対照群の参加者が意気消沈するためである。スクリーニング質問紙回答へのICと、電話CBTに関するICを求めることは、おそらく対象者のアドヒアランスを高めるだろう。

### 3.3.4. 介入への割り付け

適格性のある対象者を介入群と対照群とに1:1または1:2の割合で割り付けする。無作為割り付けは3つの変数について層別化する：ベースラインにおける抑うつ重症度(BDI2=<19 vs >=20)、ベースライン以前の絶対的presenteeism (6以上vs 5以下：パイロット研究の結果に基づく)、事業所(オフィスX、工場Y、工場Z･･･など)。対象者が3箇所からリクルートされると仮定し、合計12の層となる。本研究における統計学者は、事前に12の無作為割り付けリストを用意し、12層のうちのいずれかひとつを用いる。各リストは、ひとつの層あたり介入群対対照群の比が1:1または1:2になるようにブロック化される。統計学者は、本研究の他の業務を担当しないリサーチ・アシスタントにこのリストを送り、リサーチ・アシスタントは安全な場所でこのリストを管理し、割り付けの隠蔽化が可能なスプレッド・シートを作成する。

無作為割り付けの前に、CRCはその人が無作為化対照試験における適格性を有するかどうかの最終確認を行う。たとえば、無作為割り付けの前に、抑うつ重症度が非常に低い 人(BDI2=<9)を除外する。これは、K6とBDI2の得点に差がある場合が存在することや、スクリーニングから時間が経過していること、 ベースラインでBDI2の得点が低い人は改善の余地が少ないことによる。CRCは適格性を有する参加者のリストを作成し、各参加者がどの層に該当するかを確認し、上記スプレッド・シートに入力する。この入力はいったん行うと変更不可能である。

対照群に割り付けられた人は待機リストに載り、希望があれば割り付けから15ヶ月以降に電話CBTを受けることができる。また、同意書を返送したもののBDI2<=9となり、無作為割付の対象とならなかった参加者も、同意書の返送から15ヶ月以降に電話CBTを受けることができる。

ひとつの層別化基準はBDI2>=20 と BDI2<=19である。このカットオフは、先行研究(Hiroe et al., 2005)においてBDI2のスコアが14-19は軽症、20-28は中等症、29以上は重症であることが示唆されていること、我々のパイロット研究においてこのカットオフがK6>=9の労働者におけるBDI2の中央値とほぼ等しいことが示唆されたことにより選択された。

もうひとつの層別化基準はpresenteeism である。これは我々のプライマリ・アウトカムのひとつであるためである。また、実際にpresenteeismが影響力を持つのであるならば、おそらく障害の重い人々において何らかの影響力があることの確認が重要なためである。

# 4. 介入

## 4.1. 手順とスケジュール

無作為割り付けと介入の開始との時間間隔が可能な限り短くなるよう、CRCは2回目の同意書が返送されたその日に割り付けを行い、1回目のアポイント日時を調整し、同日にマニュアルを送付するよう努力する。

電話カウンセリングではマニュアルとクライエント・ワークブックを用いる。カウンセラー・マニュアルも用意されている。これらはすべて、“Creating a Balance: A Step-by-Step Approach to Managing Stress and Lifting Your Mood” (Simon, Ludman, & Tutty, 2006) をモデルとしたものである。これは、2つの大規模なRCTで効果が検証されているもので、プライマリ・ケア(Simon, Ludman, Tutty, Operskalski, & Von Korff, 2004)や職場(Wang et al., 2007)において薬物療法と併用されたとき、抑うつの軽減に有効であることが見出されている。

電話CBTは8セッションからなる。1セッションは導入、3セッションは行動活性化、3セッションは認知再構成、1セッションはまとめである。プログラムの内容は本研究の研究責任者たちの専門家としての考えと、パイロット研究におけるクライエントからのフィードバックをもとに改変・調整され、日本の文化における労働者の閾値下抑うつに適するものとなるようにした。各セッションでクライエントは予習をし、およそ30～45分の電話セッションを受け、ホームワークを行う。

EAPプログラムは1997から既にこの企業において用いられている。プログラムには、すべての社員が利用可能な精神的健康・身体的健康に関するホームページ、必要に応じて電話カウンセリングないし対面カウンセリングのサービス、管理職および社員に対するメンタルヘルスに関する講義が含まれている。2007年について社員1名あたりの平均サービス利用数は、web上のメンタルヘルス・セルフチェックが0.09、電話カウンセリングが0.003、対面カウンセリングが0.001となっている。この会社は社員1名あたり3ドル(約300円)を支払っている。加えて、2つの事業所には非常勤の心理士が雇われており、2006年における社員1名あたりのセッション数は0.13となっている。EAPプログラムのプロバイダーにはこの電話CBT臨床試験について伝えない。同時に、本研究の参加者には、EAPプログラムのプロバイダーと接触する際に、本臨床試験について話すことを慎むことは求めない。

## 4.2. カウンセラーのアドヒアランスの測度(クオリティ・コントロール)

介入の質を維持することは、研究の効力を最大化するために重要である。

3名のスーパーバイザーは認知療法に長けている。

3名のスーパーバイザーと9名の電話カウンセラーは、本研究で用いられている電話CBTのオリジナルの開発者であるEvette Ludman による2日間の研修、あるいはそれに準ずる研修を受けた。

心理士(修士課程大学院生、博士課程大学院生、ポスドク・レベル)は、臨床試験に入る前に、個別のスーパーバイズを1ケース分、グループ・スーパーバイズを2ケース分受けた。スーパーバイザーとカウンセラーは2ヶ月に1回の定例会議を行う。

すべてのセッション音声は録音される。セッション内容のアドヒアランスはカウンセラーのセルフチェック・リストでチェックされ、進行に沿ってCRCがさらにチェックを行う。本研究の前半・中半・後半において、録音された音声のうちランダムに10%分が抽出され、スーパーバイザーが同様のチェックリストを用いてアセスメントし、定例会議において報告され議論がなされる。独立した評価者によるアドヒアランスの程度と、自己評価と独立評価者による評価との一致の程度が報告される。

## 4.3. クライエントのアドヒアランスの測度

CRCはクライエントのアドヒアランスを確認し、以下のいずれかに該当する場合にはカウンセラーとスーパーバイザーに連絡する：

1. 割り付けから3週間以上、連絡なく経過している
2. セッション間が3週以上、連絡なく経過している
3. 第2セッション以降にK6 >=10
4. 第5セッション以降にK6 >=6

# 5. アセスメント

## 5.1. 測度

### Beck Depression Inventory-II (BDI2)

ベック抑うつ尺度のオリジナルは1961年に公刊されており(Beck, Ward, Mendelson, Mock, & Erbaugh, 1961), 抑うつ重症度の自己評価尺度として広く用いられている。DSM-IVの使用開始により,質問項目の時間的枠組みがBDI第2版で改訂され、その信頼性・妥当性が確認されている (Beck, Steer, & Brown, 1996)。日本版の信頼性と妥当性は高いことが見出されている (Hiroe et al., 2005)。

ベースライン、4ヶ月後(介入終了直後)、15ヶ月フォローアップにてBDI2を施行する。

### Health and Productivity Questionnaire (HPQ)

世界保健機構のHealth and Productivity Questionnaire (HPQ) は、健康問題による職場のコストを推定するために開発された自己報告式尺度で、自己報告による病欠(absenteeism)と仕事のパフォーマンスの低下(presenteeism)から構成されている。妥当性検証研究では、HPQで測定された労働時間と勤務記録との有意な相関(r=0.61 ～0.87, Kessler et al., 2003a)、管理者による仕事の生産性評価との有意な相関(r=0.52, Kessler et al., 2004a)、その他の事務記録との有意な相関(曲線下面積、0.58-0.72, Kessler & Ustun, 2004b)があることを見出している。

ベースライン、4ヶ月後(介入終了直後)、15ヶ月フォローアップにてHPQを施行する。

### K6

K6は近年開発された非常に短い(6項目)自己報告式質問紙で、よく見られる精神障害のスクリーニングのためのものである(Kessler et al., 2002)。 これは項目反応理論に基づくもので、一般母集団の分布の90～99パーセンタイルで、回答者の弁別力を最大にする項目から構成されている。なぜなら、どのような時点においても、5～10%の人々が精神障害を有することが知られているためである。様々な社会・人口統計学的な特徴において心理測定特性が安定して示されている項目のみが最終的に含まれている。K6はこれまでの広く用いられてきたスクリーニング質問紙と同等あるいはそれ以上に良いものであることが見出されている(Furukawa, Kessler, Slade, & Andrews, 2003; Kessler et al., 2003b)。日本語版の妥当性は既に示されている (Furukawa et al., in press)。

本研究ではK6を最初のスクリーニングとして用いる。さらに電話CBT進行中のプロセス測度として、各セッションのはじめに実施する。スクリーニングに加えて、4ヶ月後、15ヶ月フォローアップ(これらすべて統制群にも実施)で、セカンダリ・アウトカムの測度として扱われる。

### Composite International Diagnostic Interview (CIDI)

CIDIは精神障害を査定するために広く用いられている構造化診断面接で、訓練を受けた一般の面接者が一般母集団に施行するものとなっている(Robins et al., 1988)。DSM-IV に合わせて改訂がなされている。我々は最新のコンピュータ化された版を用い(Kessler et al., 2004b)、気分障害およびアルコール関連のセクションを実施する。CIDIと標準化された臨床的アセスメントとの一致は既に報告されている (Haro et al., 2006)。 HPQとCIDIの日本語版は日本におけるWorld Mental Health Survey で使用されている (Kawakami et al., 2005)。

## 5.2. フォローアップの手順とスケジュール

### 5.2.1. 介入からの脱落

参加者はいつでも介入および(または)研究への参加を辞退することができる。しかしながら、カウンセラーとCRCは、介入から脱落した人に4ヶ月後のフォローアップ調査を依頼することがいかに重要であるかということを認識するようにしなければならない。もし介入への参加を中断するとしても、4ヶ月後および15ヶ月後のフォローアップでアウトカム・データを収集するあらゆる努力をしなければならない。新たな精神症状が生じたり、精神症状の増悪が生じた場合には、電話CBTの実施中であってもそうでなくても、すべての参加者は企業から提供されているEAPサービスを通常どおり使用したり、企業外部の専門的援助を求めることができる。さらに、研究センターの心理士につながる通話料無料のホットラインに電話をかけることができる。本プログラム以外の治療が望ましいと考えられるような、新たな精神症状の発生、現存する閾値下抑うつの増悪等が電話CBT中に見られた場合には、カウンセラーは参加者に対して、産業医、かかりつけ医、ないしメンタルヘルスの専門家に直接相談することを勧めなければならない。カウンセラーと参加者が、電話CBTと新たに勧められたその他の治療との間に葛藤を感じなければ、介入への参加は継続され、もしなんらかの葛藤を感じたり、参加者の側から介入の辞退の要望があった場合には中止しなければならない。電話CBTが中止となった場合でも、参加者は4ヶ月後と15ヶ月後のフォローアップには参加するよう促される。

### 5.2.2. 研究からの脱落

4ヶ月後および15ヶ月後のフォローアップに対して同意を撤回した参加者は、研究からの脱落とみなされる。

### 5.2.3. どのようにアドヒアランスを高め、脱落を減らすか

介入からの脱落は研究からの脱落とは厳密に区別される。クライエントには。電話カウンセリングを中止したとしても、可能な限り最終的なアセスメントへの回答を依頼する。

ICでの説明では、アドヒアランスを高めるために、スクリーニングのデータから明らかになったクライエントの感受性・脆弱性について、プログラムから得られると考えられる利益について強調する。

プログラムの説明にはパンフレットを用いる。

ICを2回とることは、不熱心な参加者を除き、熱心な参加者のアドヒアランスを高めるだろう。

2010年には、介入群・対照群の双方に年賀状を送付するが、これは第1コホートの15ヶ月フォローアップの直前となる。2011年にも送付する。4ヶ月・15ヶ月フォローアップの後には、御礼カードを送付する。特に4ヶ月(プライマリ・アウトカム)と15ヶ月後フォローアップでは、謝金その他の報酬を与えられれば良いが、これにはスポンサーとの交渉が必要である。

## 5.3. 反応測度のアセスメント

### 5.3.1. 自己報告式質問紙の独立性

プライマリ・アウトカムであるBDI2(参加者が回答)は、カウンセラーではなくCRC宛で厳封した返送されるが、これは評価の匿名性と独立性を保つためである。

添付の手紙では、データをカウンセラーが見ることはないことをはっきりと説明し、正直に回答することが望まれることを伝える。

### 5.3.2. データ収集

#### 4ヶ月フォローアップ (介入直後のアウトカム)

介入群においては、プライマリ・アウトカムは無作為割付けから4ヶ月後(電話カウンセリングの最終セッションから約1ヶ月後)に測定される。

対照群では、無作為割り付けから4ヶ月の時点でエンドポイントのアセスメントが行われる。

質問紙は参加者に郵送される。2週間以内に返送がない場合には、カバーレターを添えて、再度質問紙を送付する。それでも返送がない場合には、CRCは参加者(介入群・対照群いずれも)に電話をする。質問紙に対する電話での回答は許容範囲とする。

#### 15ヶ月フォローアップ

介入群・対照群のいずれも、無作為割り付けから15ヶ月後に質問紙を受け取る。

CRCは質問紙を実際に送付する2週間前に、ポストカードを送付する(注意喚起のため)。その後、質問紙が参加者に送られる。2週間以内に返送がない場合には、カバーレターを添えて、再度質問紙を送付する。それでも返送がない場合には、CRCは参加者(介入群・対照群いずれも)に電話をする。

### 5.3.3. CIDI面接者の研修

CIDIは、事前に研修を受け経験を積んだ2名の面接者と、研修を受け数多くの面接経験を持つ3名の面接者によって施行される。

CIDI以外の回答はすべて自己報告である。

### 5.3.4. クオリティー・コントロール

ベースラインと15ヶ月フォローアップで行われるすべてのCIDI面接は録音され、ランダムに抽出された10%分が評定者間信頼性の検討のために確認される。CRCはスクリーニング(2)におけるCIDI面接の約半分を担当するが、15ヶ月後のCIDIの担当からは外れるため、割り付け後の評価の独立性は保たれる。

CRCはすべての自己報告をチェックし、欠損がある場合には参加者に尋ねる。

# 6. 解析

## 6.1. データ解析

### 6.1.1. 中間モニタリング

中間解析は行われない。なぜなら、最初の9ヶ月はリクルートが順に進められているということと、4ヶ月フォローアップのアウトカム・データを得るまでに、予定しているサンプルサイズの半数はリクルート完了となる予定となっているためである。

### 6.1.2. 予備的解析

解析の第1歩は、想定される範囲外の値と、論理的に矛盾する部分(過去1ヶ月に休んだ日はないと回答しておきながら、後続する質問で4日間病欠だったと回答するというような場合がある)をチェックすることである。

各アウトカム変数において要約統計量(平均、標準偏差、最小値、最大値、歪度)を算出し、各アウトカムの分布図を得るために、ヒストグラム・プロットを行う。これをベースライン、4ヶ月後、15ヶ月後のそれぞれについて行う。

### 6.1.3. ベースライン・データの解析

BDI2やHPQといった連続変数や順序変数については、対照群と介入群の平均値を求め、t検定により群間に有意差があるかどうかを検証する。ベースラインのすべての2値変数(はい/いいえ)については、2群において「はい」である割合を求め、自由度1のχ^2^検定を行う。ベースラインの群間比較を行う“Table 1”では、p値を報告するかもしれないし、報告しないかもしれない。投稿する雑誌によって報告を求めるところとそうでないところがあるため、p値を報告するか否かは投稿する雑誌による。

### 6.1.4. プライマリ・アウトカムの解析

2つのプライマリ・アウトカム、抑うつ重症度の測度であるBDI2と、有効労働時間の測度であるHPQは、どちらも4ヶ月フォローアップで測定される。これらはどちらも連続変数/順序変数であるため、対照群と介入群の2群で、BDI2とHPQそれぞれの平均値を算出する。

まずはじめに平均値を比較するため、t検定を用いて調整なしの解析を行う。その後、いくつかの調整解析を行う。第1には、ベースラインの差を調整して、4ヶ月フォローアップの平均値を比較するために、ANCOVAを行う。ANCOVAは回帰分析の一種である。たとえば、あるモデルにおいて、4ヶ月後のBDI2がアウトカムとなり、群(対照群/介入群の2値)とベースラインのBDI2の値が独立変数となる。これらのANCOVAは、4ヶ月時点での調整済み群平均を、その群内平均の調整済みの差の推定値(p値と信頼区間を併せて)とともに示すものである。

また、その他のベースラインの変数を統制する調整解析も同様に行うが、統制群と介入群との間で有意な差がある変数と、解析対象となるアウトカムの強い予測因子となる変数を含む。群内の差の推定値とp値を、調整なしの解析結果と、アウトカムのベースラインの値のみ調整したANCOVAの結果とを比較する。

これらの解析は、4ヶ月フォローアップを完了した対象者に限られる(上記の統計解析方法ではすべて、アウトカムについて欠損値のある対象者は解析対象外となる)。真のintent-to-treat分析では、無作為割り付けされたすべての対象者のデータをもとに治療効果を推定するのであって、フォローアップを完了した対象者のみを対象とするものではないが、すべての対象者についての治療効果を推定するために、階層的線形(混合)モデル(HLM)による分析を行う。注）4ヶ月フォローアップの回答率が高い場合には(最低90%)、欠損値によるバイアスは非常に小さいと考えられるので、これらの解析は不要であろう。

HLM分析が必要な場合、混合ランダム係数モデルと呼ばれるものを用いる。対象者は、様々な切片(ベースラインの値)と様々な傾き(ベースラインから4ヶ月への変化率)を取るランダム要因となる。データセットは対象者1名あたり2つのレコードから構成される：ひとつはベースライン、もうひとつは4ヶ月フォローアップ。ベースラインでしか回答しなかった対象者も解析に含められ、各群の4ヶ月後の平均値の推定において考慮される。たとえば、4ヶ月フォローアップを完了しなかった対象者は、完了した対象者よりも、ベースラインのBDI2スコアが高かったとすると、解析における4ヶ月フォローアップにおける平均の推定値は、先述の回答者のみの解析の場合よりも高くなる。 このようなモデルはSASやSTATA、SPSSといった標準的な統計パッケージで適用可能である。

たとえば、BDI2のランダム係数ANCOVAは以下の式からなる：

BD12_month4_ = 切片 + β_1_*treatment + β_2_ * BD12_baseline_

切片とβ_2_はランダム要因としてモデル化されている。β_1_は固定効果であり、介入群と対照群における平均値の差を推定する(治療効果の推定値)。

回答者のみの解析に基づく結論と、intent-to-treat 解析に基づく結論が異なっていた場合には、intent-to-treat 解析の結果を報告する。

### 6.1.5. セカンダリ・アウトカムの解析

連続変数/順序変数のアウトカムも、上記と同様の方法で扱われる：t検定により群平均を調整なしで比較し、その後、アウトカムのベースライン値を調整したANCOVAによる群平均の比較を行い、最後にその他のベースライン変数を調整した回帰分析を行う。 4ヶ月フォローアップの回答率によっては(そして回答率が対照群と介入群で等しい場合には)、群平均を階層的線形(混合)モデル(HLM)を用いて比較する。

フォローアップまでに仕事を辞めたかどうかというような二値変数(はい/いいえ)については、対照群と介入群の「はい」の割合を比較するが、まずは調整なしの解析を行うためにχ^2^検定を用いる。その後、アウトカム変数のベースラインのみを調整したANCOVAタイプの解析を行うために、ロジスティック回帰分析を行う。最後に、他のベースライン変数を統制した調整ロジスティック回帰分析を行う。

### 6.1.5. 研究からの脱落についての解析

4ヶ月フォローアップ完了者と非完了者との間で、ベースラインにおける特性を比較する。4ヶ月フォローアップで回答しなかった対象者が、何か重要な傾向を以て回答した対象者と違いがあるのかどうかが知りたいところである。たとえば、非完了者がベースラインにおいてより重症であるかどうかについて知ることは重要である。もしそうであるならば、回答率が対照群と介入群との間で異なるとき特に、研究からの脱落は結果にバイアスをもたらすだろう。

Table 1のすべての変数と、プライマリ・アウトカム、セカンダリ・アウトカムについて、フォローアップ完了者と非完了者の割合を算出する。完了者と非完了者における、連続変数/順序変数の平均値を比較するためにt検定を用いる。二値変数(はい/いいえ)については、自由度1のχ^2^検定において、両者の割合を比較する。

## 6.2. 臨床試験終了の方針

リクルートは9ヶ月間(12ヶ月まで延長可能)を予定している。その後、1年後のフォローアップが開始となるが、治療群・待機群ともに無作為割り付けから15ヶ月後のフォローアップとなる。

この15ヶ月フォローアップ評価の後、待機群で電話CBTを希望する対象者は、電話CBTを受けることができる。この追加CBTについては、付加的な研究となる。

# 7. 組織

## 7.1. プロジェクト・メンバー

### 7.1.1. プロジェクト管理チーム

#### 研究責任者

古川壽亮，Toshi A. Furukawa, MD, PhD (精神科医)

堀越　勝, PhD (臨床心理士)

川上憲人, MD, MPH (精神保健疫学者)

#### 企業代表者

東儀彰子, PhD

多田直哉, MSc

#### 臨床研究コーディネーター

佐々木恵, PhD (臨床心理士)

### 7.1.2. データ管理センター

名古屋市立大学大学院医学研究科精神・認知・行動医学分野に設置されたデータ管理センターには佐々木恵のみが属する。

### 7.1.3. カウンセラーとスーパーバイザー

#### スーパーバイザー

古川壽亮

堀越　勝

堀越あゆみ

#### CIDI面接者

佐々木恵(CRC兼任、CIDIの研修受講済み)

北川砂織(CIDIの研修受講済み であり、精神医学に関する疫学調査の参加経験がある)

土屋政雄(CIDIの研修受講済み、博士後期課程大学院生)

島田恭子(CIDIの研修受講済み、博士前期課程大学院生)

大江悠樹(CIDIの研修受講済み、博士前期課程大学院生)

#### 電話カウンセラー

福森崇貴(臨床心理士、PhD)

寺島　瞳(臨床心理士、PhD)

永作　稔(臨床心理士、PhD)

樫村正美(臨床心理士、MSc)

細越寛樹(臨床心理士、PhD)

丹羽まどか(臨床心理士、MSc、博士後期課程大学院生)

関屋裕希(博士前期課程大学院生)

浅野憲一(MSc、博士後期課程大学院生)

岩佐和典(MSc、博士後期課程大学院生)

### 7.1.4. 統計解析部門

古川壽亮が統計解析についての責任を担う。

Mr Louis Grothausは、研究デザインや実施、どのように解析を行い、どのように結果を解釈すべきかについて、また、公刊に向けての結果の報告について、古川と協議する。

## 8.2.研究の実施

### 8.2.1. 運営委員会と下位委員会

プロジェクト管理チームは進捗を確認するため、2ヶ月ごとに打ち合わせを行う。

カウンセラーの動機づけを高めるため、研究の進捗状況は、2ヶ月に1回の打ち合わせにてカウンセラーに報告される。

### 8.2.2. データ・安全管理委員会(Data and Safety Monitoring Board :DSMB)

DSMBはCRC、 Mr. Louis Grothaus( 統計コンサルタント)、企業代表者2名および臨床試験に慣れている古川、本研究には関与していない者(検討中)から構成される。

DSMBは最初のクライエントが無作為割り付けされてから6ヶ月ごとに打ち合わせを行う。打ち合わせの目的はCRCが同意により用意された報告を確認することである。

CRCはリクルート状況、データ収集状況(回収率)、有害事象およびその対応方法について確認するための報告を行う。

自殺念慮その他、抑うつの増悪が生じた場合を想定し、参加者が利用できるフリーの電話番号を用意する。

### 8.2.3. 財務組織

積水化学工業(株)

# 文献

Allart-van Dam, E., Hosman, C. M., Hoogduin, C. A., & Schaap, C. P. (2007). Prevention of depression in subclinically depressed adults: follow-up effects on the 'Coping with Depression' course. *Journal of Affective Disorders* **97,** 219-28.

Beck, A. T., Steer, R. A., & Brown, G. K. (1996). *BDI-II: Beck Depression Inventory, Second Edition, Manual*. San Antonia: The Psychological Corporation.

Beck, A. T., Ward, C. H., Mendelson, M., Mock, J., & Erbaugh, J. (1961). An inventory for measuring depression. *Archives of General Psychiatry* **4,** 561-71.

Broadhead, W. E., Blazer, D. G., George, L. K., & Tse, C. K. (1990). Depression, disability days, and days lost from work in a prospective epidemiologic survey. *JAMA* **264,** 2524-8.

Clarke, G. N., Hawkins, W., Murphy, M., Sheeber, L. B., Lewinsohn, P. M., & Seeley, J. R. (1995). Targeted prevention of unipolar depressive disorder in an at-risk sample of high school adolescents: a randomized trial of a group cognitive intervention. *Journal of the American Academy of Child and Adolescent Psychiatry* **34,** 312-21.

Clarke, G. N., Hornbrook, M., Lynch, F., Polen, M., Gale, J., Beardslee, W., O'Connor, E., & Seeley, J. (2001). A randomized trial of a group cognitive intervention for preventing depression in adolescent offspring of depressed parents. *Archives of General Psychiatry* **58,** 1127-34.

Cuijpers, P., Smit, F., & van Straten, A. (2007). Psychological treatments of subthreshold depression: a meta-analytic review. *Acta Psychiatrica Scandinavica* **115,** 434-41.

Dimidjian, S., Hollon, S. D., Dobson, K. S., Schmaling, K. B., Kohlenberg, R. J., Addis, M. E., Gallop, R., McGlinchey, J. B., Markley, D. K., Gollan, J. K., Atkins, D. C., Dunner, D. L., & Jacobson, N. S. (2006). Randomized trial of behavioral activation, cognitive therapy, and antidepressant medication in the acute treatment of adults with major depression. *Journal of Consulting and Clinical Psychology* **74,** 658-70.

Druss, B. G., Marcus, S. C., Olfson, M., Tanielian, T., Elinson, L., & Pincus, H. A. (2001). Comparing the national economic burden of five chronic conditions. *Health Affairs* **20,** 233-41.

Furukawa, T. A., Kawakami, N., Saitoh, M., Ono, Y., Nakane, Y., Nakamura, Y., Tachimori, H., Iwata, N., Uda, H., Nakane, H., Watanabe, M., Naganuma, Y., Hata, Y., Kobayashi, M., & Miyake, Y. (in press). The performance of the Japanese version of the K6 and K10 in the World Mental Health Survey Japan. *International Journal of Methods in Psychiatric Research*.

Furukawa, T. A., Kessler, R. C., Slade, T., & Andrews, G. (2003). The performance of the K6 and K10 screening scales for psychological distress in the Australian National Survey of Mental Health and Well-Being. *Psychol Med* **33,** 357-62.

Geddes, J. R., Carney, S. M., Davies, C., Furukawa, T. A., Kupfer, D. J., Frank, E., & Goodwin, G. M. (2003). Relapse prevention with antidepressant drug treatment in depressive disorders: a systematic review. *Lancet* **361,** 653-61.

Greenberg, P. E., Kessler, R. C., Birnbaum, H. G., Leong, S. A., Lowe, S. W., Berglund, P. A., & Corey-Lisle, P. K. (2003). The economic burden of depression in the United States: how did it change between 1990 and 2000? *Journal of Clinical Psychiatry* **64,** 1465-75.

Haro, J. M., Arbabzadeh-Bouchez, S., Brugha, T. S., de Girolamo, G., Guyer, M. E., Jin, R., Lepine, J. P., Mazzi, F., Reneses, B., Vilagut, G., Sampson, N. A., & Kessler, R. C. (2006). Concordance of the Composite International Diagnostic Interview Version 3.0 (CIDI 3.0) with standardized clinical assessments in the WHO World Mental Health surveys. *International Journal of Methods in Psychiatric Research* **15,** 167-80.

Hiroe, T., Kojima, M., Yamamoto, I., Nojima, S., Kinoshita, Y., Hashimoto, N., Watanabe, N., Maeda, T., & Furukawa, T. A. (2005). Gradations of clinical severity and sensitivity to change assessed with the Beck Depression Inventory-II in Japanese patients with depression. *Psychiatry Research* **135,** 229-35.

Judd, L. L., Akiskal, H. S., & Paulus, M. P. (1997). The role and clinical significance of subsyndromal depressive symptoms (SSD) in unipolar major depressive disorder. *Journal of Affective Disorders* **45,** 5-17; discussion 17-8.

Judd, L. L., Schettler, P. J., & Akiskal, H. S. (2002). The prevalence, clinical relevance, and public health significance of subthreshold depressions. *Psychiatric Clinics of North America* **25,** 685-98.

Kawakami, N., Takeshima, T., Ono, Y., Uda, H., Hata, Y., Nakane, Y., Nakane, H., Iwata, N., Furukawa, T. A., & Kikkawa, T. (2005). Twelve-month prevalence, severity, and treatment of common mental disorders in communities in Japan: preliminary finding from the World Mental Health Japan Survey 2002-2003. *Psychiatry and Clinical Neurosciences* **59,** 441-52.

Kessler, R. C., Akiskal, H. S., Ames, M., Birnbaum, H., Greenberg, P., Hirschfeld, R. M., Jin, R., Merikangas, K. R., Simon, G. E., & Wang, P. S. (2006). Prevalence and effects of mood disorders on work performance in a nationally representative sample of U.S. workers. *American Journal of Psychiatry* **163,** 1561-8.

Kessler, R. C., Ames, M., Hymel, P. A., Loeppke, R., McKenas, D. K., Richling, D. E., Stang, P. E., & Ustun, T. B. (2004a). Using the World Health Organization Health and Work Performance Questionnaire (HPQ) to evaluate the indirect workplace costs of illness. *Journal of Occupational and Environmental Medicine* **46,** S23-37.

Kessler, R. C., Andrews, G., Colpe, L. J., Hiripi, E., Mroczek, D. K., Normand, S. L., Walters, E. E., & Zaslavsky, A. M. (2002). Short screening scales to monitor population prevalences and trends in non-specific psychological distress. *Psychol Med* **32,** 959-76.

Kessler, R. C., Barber, C., Beck, A., Berglund, P., Cleary, P. D., McKenas, D., Pronk, N., Simon, G., Stang, P., Ustun, T. B., & Wang, P. (2003a). The World Health Organization Health and Work Performance Questionnaire (HPQ). *Journal of Occupational and Environmental Medicine* **45,** 156-74.

Kessler, R. C., Barker, P. R., Colpe, L. J., Epstein, J. F., Gfroerer, J. C., Hiripi, E., Howes, M. J., Normand, S. L., Manderscheid, R. W., Walters, E. E., & Zaslavsky, A. M. (2003b). Screening for serious mental illness in the general population. *Archives of General Psychiatry* **60,** 184-9.

Kessler, R. C., & Ustun, T. B. (2004b). The World Mental Health (WMH) Survey Initiative Version of the World Health Organization (WHO) Composite International Diagnostic Interview (CIDI). *International Journal of Methods in Psychiatric Research* **13,** 93-121.

Paykel, E. S., Freeling, P., & Hollyman, J. A. (1988). Are tricyclic antidepressants useful for mild depression? A placebo controlled trial. *Pharmacopsychiatry* **21,** 15-8.

Richardson, K. M., & Rothstein, H. R. (2008). Effects of occupational stress management intervention programs: A meta-analysis. *Journal of Occupational Health Psychology* **13,** 69-93.

Robins, L. N., Wing, J., Wittchen, H. U., Helzer, J. E., Babor, T. F., Burke, J., Farmer, A., Jablenski, A., Pickens, R., Regier, D. A., & et al. (1988). The Composite International Diagnostic Interview. An epidemiologic Instrument suitable for use in conjunction with different diagnostic systems and in different cultures. *Archives of General Psychiatry* **45,** 1069-77.

Rost, K., Smith, J. L., & Dickinson, M. (2004). The effect of improving primary care depression management on employee absenteeism and productivity. A randomized trial. *Medical Care* **42,** 1202-10.

Schoenbaum, M., Kelleher, K., Lave, J. R., Green, S., Keyser, D., & Pincus, H. (2004). Exploratory evidence on the market for effective depression care in Pittsburgh. *Psychiatric Services* **55,** 392-5.

Simon, G. E., Ludman, E. J., & Tutty, S. (2006). *Creating a Balance: A Step-by-Step Approach to Managing Stress and Lifting Your Mood*. Victoria, British Columbia, Canada: Trafford Publishing.

Simon, G. E., Ludman, E. J., Tutty, S., Operskalski, B., & Von Korff, M. (2004). Telephone psychotherapy and telephone care management for primary care patients starting antidepressant treatment: a randomized controlled trial. *JAMA* **292,** 935-42.

Spek, V., Nyklicek, I., Smits, N., Cuijpers, P., Riper, H., Keyzer, J., & Pop, V. (2007). Internet-based cognitive behavioural therapy for subthreshold depression in people over 50 years old: a randomized controlled clinical trial. *Psychol Med* **37,** 1797-806.

Stewart, W. F., Ricci, J. A., Chee, E., Hahn, S. R., & Morganstein, D. (2003). Cost of lost productive work time among US workers with depression. *JAMA* **289,** 3135-44.

van der Klink, J. J., Blonk, R. W., Schene, A. H., & van Dijk, F. J. (2001). The benefits of interventions for work-related stress. *American Journal of Public Health* **91,** 270-6.

Wang, P. S., Beck, A., Berglund, P., Leutzinger, J. A., Pronk, N., Richling, D., Schenk, T. W., Simon, G., Stang, P., Ustun, T. B., & Kessler, R. C. (2003). Chronic medical conditions and work performance in the health and work performance questionnaire calibration surveys. *Journal of Occupational and Environmental Medicine* **45,** 1303-11.

Wang, P. S., Simon, G. E., Avorn, J., Azocar, F., Ludman, E. J., McCulloch, J., Petukhova, M. Z., & Kessler, R. C. (2007). Telephone screening, outreach, and care management for depressed workers and impact on clinical and work productivity outcomes: a randomized controlled trial. *JAMA* **298,** 1401-11.

Wells, K. B., Stewart, A., Hays, R. D., Burnam, M. A., Rogers, W., Daniels, M., Berry, S., Greenfield, S., & Ware, J. (1989). The functioning and well-being of depressed patients. Results from the Medical Outcomes Study. *JAMA* **262,** 914-9.

Willemse, G. R., Smit, F., Cuijpers, P., & Tiemens, B. G. (2004). Minimal-contact psychotherapy for sub-threshold depression in primary care. Randomised trial. *British Journal of Psychiatry* **185,** 416-21.

World Health Organization. (2004). World Health Report 2004.
