# Supplementary material for: Telephone Cognitive-Behavioral Therapy for Subthreshold Depression and Presenteeism in Workplace: A Randomized Controlled Trial
Source: PLoS One. 2012 Apr 19;7(4):e35330. doi: 10.1371/journal.pone.0035330 (PMC3330821; doi:10.1371/journal.pone.0035330)
Supplement: Protocol S2 — English translation of the original Japanese protocol. (DOCX) [file pone.0035330.s003.docx]

Telephone cognitive-behavioral therapy for subthreshold depression and pressenteeism in workplace: a randomized controlled trial

Toshi A. Furukawa, Masaru Horikoshi, Norito Kawakami, Megumi Sasaki, Ayumi Horikoshi, [counselors who have seen a substantial number of clients], Masao Tsuchiya, Lou Grothaus

This trial has been registered at clinicaltrials.gov (Identifier: NCT-XXX).

# 1. BACKGROUND

Depression is costly for the individual and for the society world-wide and especially in established market-economy countries (World Health Organization, 2004). In monetary terms, the cost of depression due to treatments, reduced productivity and suicide has been estimated to amount to $83 billion for the year 2000 in the USA alone (Greenberg et al., 2003). This societal cost is only second to that of hypertension and is indeed far greater than that of other chronic conditions such as diabetes, heart disease or asthma (Druss et al., 2001).

Of note is the fact that the direct treatment costs account for only about 25% of the total societal cost of depression. As a matter of fact, the great majority of the cost of depression ($52 billion, or 62%) is incurred in the workplace as lost productive work time (Greenberg et al., 2003). Other researchers using different methodology have arrived at comparable estimates; for example, according to (Druss et al., 2001) cost of work loss among those who were treated for depression would be $11.5 billion for the whole USA in 1996. If all workers of depression, regardless of their treatment status, were counted, the sum went up to $44 billion in another study (Stewart et al., 2003). The estimates according to the National Comorbidity Survey Replication amounted to $36.6 billion (Kessler et al., 2006).

Of further note is the fact that the cost of depression in the workplace is not mainly due to absence from the job (absenteeism) but rather from reduced productivity while on the job (presenteeism). For example, the American Productivity Audit estimated hours lost due to depression among workers with major depression were 1.2 hours/week for absenteeism and 7.2 hours/week for presenteeism (Stewart et al., 2003). A newer survey revealed that depression was responsible for 8.7 days equivalent of absenteeism and 18.2 days equivalent of presenteeism per year per depressed employee (Kessler et al., 2006). Depression is indeed among the most costly of all health problems to employers (Wang et al., 2003). From a more holistic viewpoint, it is important to remember here that all this money counting has not yet taken into account the personal subjective sufferings of the afflicted and those of the family.

It is then no wonder then that several groups of researchers have sought for ways to reduce depression’s deleterious effects in the workplace. While a good number of controlled trials are reported in the literature that tried to reduce work-related stress in general through organizational, cognitive-behavioral and other interventions (Richardson and Rothstein, 2008; van der Klink et al., 2001), only two trials have examined work-related productivity loss. Rost et al (Rost et al., 2004) compared enhanced care against usual care for workers visiting twelve community primary care practices in USA. The enhanced care involved, in addition to the usual care by the primary care physicians, telephone contacts by care managers who reassessed the patients’ depressive symptoms, educated them about depression and its treatment, reported the patients’ status to the physicians and made recommendations for adjustment of treatment in accordance with the treatment guideline. Over two years, the intervention had some limited impact on depression (p=0.09) but significantly improved productivity by 6.1% (p<0.05) and tended to improve absenteeism by 22.8% (p=0.06). In monetary terms, the gain translated into $1491 for decreased presenteeism and $539 for absenteeism per one depressed employee per annum. These effects were more salient among consistently employed subjects.

Wang et al (Wang et al., 2007) examined a more aggressive outreach-treatment program in the workplace recently. They first screened employees from 16 large companies from diverse sectors and those who screened positive for possible depression were randomly allocated to usual care or intervention. Those assigned to usual care were informed that their responses indicated possible depression and advised to consult with a clinician. Those assigned to intervention received a systematic telephone intervention program, which assessed needs for treatment, facilitated entry into in-person treatment, monitored and supported treatment adherence and, for those who declined in-person treatment, provided a structured telephone psychotherapy. The intervention not only significantly reduced depression severity (p=0.001) but also increased effective hours worked (p=0.002) and job retention (p=0.02) through 12 months. The gain translated into 2 more hours per week, or 2 more weeks per year, than workers assigned to the usual care.

What we should not lose sight of in our fight against lost productivity in workplace is that minor depression or subthreshold depression, although less symptomatic than full major depression by definition, is still associated with substantial functional impairment (Judd et al., 2002; Wells et al., 1989) and as a matter of fact, due to its greater prevalence, may be responsible for twice as many disability days in toto than major depression per se (Broadhead et al., 1990). Moreover, minor depression may well be the most ominous harbinger to subsequent full-blown major depression (Judd et al., 1997).

Fortunately there is growing evidence that psychotherapies, especially cognitive-behavioral ones, may not only ameliorate subthreshold depression (Allart-van Dam et al., 2007; Cuijpers et al., 2007; Spek et al., 2007; Willemse et al., 2004) but also may prevent future major depressive episodes up to 18 months (Clarke et al., 1995; Clarke et al., 2001). On the other hand available evidence does not support effectiveness of pharmacotherapy for minor depression (Dimidjian et al., 2006; Paykel et al., 1988), except in the context of relapse prevention for recurrent depression (Geddes et al., 2003). In this randomized controlled study we therefore aimed:

1. To demonstrate the effectiveness of the CBT package for subtheshold depression in the workplace
2. To demonstrate the economic worthiness of this CBT package in the workplace

Correspondingly, the hypotheses addressed in this study include:

1. Whether the CBT package is significantly more effective than the usual EAP package in reducing the depression severity and preventing depression episodes among workers with subthreshold depression
2. Whether the CBT package can enhance work performance by at least $100 on average per worker per annum
   1. If all this is to be gained by increased work performance (greater effective hours worked), among 100 workers with an average hourly wage of $40, 10 must receive the package and gain 2 effective hours worked per month to compensate for $10000 per year.
   2. And if the CBT program prevents 0.1 person from resigning, this will mean a gain of $8000 per annum in terms of yearly wage, in addition to the educational and other costs that the company has poured into the workers.

The study will be a two-arm, parallel-group, TAU-controlled, non-blinded randomized study.

# 2. OBJECTIVES

## 2.1 Primary question

Our primary question is: “Among company employees with subthreshold depression, will a telephone-CBT program consisting of behavioral activation (BA) and cognitive restructuring (CR) in addition to the standard Employee Assistance Program (EAP) outperform EAP alone, in terms of depression severity (measured with BDI2) and work productivity (measured with HPQ) at the end of the program?”

The primary outcome measures in this study will therefore be:

1) depression severity at 4 months post-randomization as measured by the BD12

2) work performance at 4 months post-randomization as measured by the HPQ, which assesses “effective hours worked” for the past month

These two primary outcomes will be used to decide whether or not the intervention was effective.

## 2.2. Secondary questions

Secondary outcomes will include:

1. At 4 months post-randomization
   1. depression severity as measured by K6
   2. client satisfaction　[VAS for satisfaction with the company’s support for mental stress, and a detailed ad hoc questionnaire for those who received telephone CBT]
2. At 15 months post-randomization
   1. depression severity [BD12 and K6]
   2. work performance
      1. “Effective hours worked” for the past month [HPQ]
      2. Job retention: has the person quit his/her job by 15 months post-randomization [yes/no, if yes when did the participant quit the job? The latter information is important for survival analysis.]
   3. client satisfaction [VAS]
   4. use of EAP services, use of health services (physical or mental), suicidal attempt, hospitalization (physical or mental) [yes/no for 15 months post randomization]
   5. time to incident major or minor depressive episode during the 15 months post randomization

## 2.3. Subgroup hypotheses

The effectiveness of the program may differ according to the initial severity of depression. We will therefore use as one stratification factor high/low subthreshold depression, and analyze the results according to these a priori defined subgroups. Because we do not have enough sample size to have adequate power to detect if treatment effects vary significantly by subgroup (i.e. for subgroup hypotheses), these will be more exploratory (i.e. hypothesis generating) than definitive analyses. Although we will test for treatment by subgroup interactions, our focus will be descriptive, describing the pattern of treatment effects by subgroup. To reduce the number of these analyses, we will focus on our primary outcomes (BD12 and HPQ at month 4).

## 2.4. Adverse effects

We expect no adverse health effect from this intervention, except possibly for deterioration in depression (and, ultimately, suicide). We will provide the emergency phone call number at the central office, should any serious suicidal wish occurs. The clinical research coordinator (CRC), who is a clinical psychologist (MS), will then deal with the emergency call first by herself, and then consult with the telephone counselor in charge and/or the clinical supervisors (TAF, MH, AH) to provide appropriate care. Any indirect information regarding depressive deterioration and/or suicidal ideation/attempts from any source will be fed into the CRC and will be discussed with the clinical supervisors and the individually and clinically most appropriate course of action will be taken.

We will measure deterioration in depression in our primary and secondary outcomes. We will measure suicidal attempt, completed suicide and hospitalization at 15-month follow-up. We will not implement “stopping decision rules” for this study, because we will not have a sample large enough to determine whether adverse events are clearly related to study participation or if outcome data are sufficient to answer our primary study questions prior to enrollment of the full sample. Professor Furukawa, the Principal Investigator, will be responsible for all the decisions. Professor Furukawa will be available for review of all instances of reported adverse effects and suicide risk within 24 hours. When he is not available to fulfill this role because of travel or other obligations, Professor Horikoshi or another designated clinical investigator will be appointed to monitor participant safety.

# 3. PARTICIPANTS

## 3.1. Study population

Working men and women with subthreshold depression will be selected according to the following criteria:

### 3.1.1. Inclusion criteria

1. Age 20-57 at study entry [because the retirement age is usually 60, we have to recruit the patients well before 60 in order to allow time for follow-up]
2. Men and women
3. Currently employed full-time (either regular or temporary) by the business company
4. Expected to be employed full-time for 2 years after screening
5. K6 scores greater than or equal to 9 (or 10) at screening
6. BDI2 scores greater than or equal to 10 at 2^nd^ screening (CIDI interview)

### 3.1.2. Exclusion criteria

1. Part-time employees
2. Sick leave for 6 or more days for a physical or mental condition in the past month
3. Expected to be on pregnancy leave, maternity leave or nursing leave within 2 years after screening
4. Current treatment for a mental health problem from a mental health professional
5. Major depressive episode in the past month, as ascertained by CIDI [We do not exclude dysthymia or major depression in partial remission]
6. Lifetime history of bipolar disorder, as ascertained by CIDI
7. Any substance dependence in the past 12 months, as ascertained by CIDI. [We do not exclude substance abuse.]
8. Any other current mental disorder if it constitutes the predominant aspect of the clinical presentation and requires treatment not offered in the project

We will have a registry of all the candidates at Office X and Factory Y (approximately 1200 regular or temporary employees altogether) of this company to whom we will send the invitation letter, and compare those who give the full consent to participate in the intervention trial to the original population, to examine the generalizability of our results.

## 3.2. Sample size assumptions and estimates

A systematic review of psychological treatments, mostly CBT, for subthreshold depression yielded a Cohen’s d of 0.42 (95%CI: 0.23 to 0.60) at post-test and 0.16 (95%CI: 0.02 to 0.35) at 1-year follow-up (Cuijpers et al., 2007). ( a Cohen's d of .42 indicates a treatment effect (difference between control mean and intervention mean on the outcome measure) equal to 0.42 standard deviations (where standard deviation(SD) of interest if the SD of the outcome measure)

|  |  |  | 1:1 |  | If 1:2 |  | If 1:3 |  |
| --- | --- | --- | --- | --- | --- | --- | --- | --- |
| alpha | power | Effect size | n in each cell | Total N | n in active treatment | Total N | n in active treatment | Total N |
| 0.05 | 0.90 | 0.4 | **131** | 262 | **99** | 297 | **88** | 350 |
| 0.05 | 0.80 | 0.4 | **98** | 196 |  |  |  |  |

Thus, if we randomize 99 in the intervention group and twice that many in the control group (198) for a total of 297 randomized, we will have 90% power to detect a treatment effect assuming that this difference is equal to .40 standard deviations. However, these calculations ignore dropout. We expect that 90% will complete our month 4 followup, resulting in 89 respondents in the intervention group and 178 respondents at month 4 in the control group. In this situation, we will have 90% power to detect an effect size equal to .42 SD, which is exactly equal to our best estimate of what effect we expect to see at month 4 in this trial.

At month 15, we will have inadequate power to detect treatment effects since we expect those effects to be much smaller than those seen at month 4 (only about .16 SD's). However, in order to obtain insight into the next RCT to demonstrate prophylactic effect, we will conduct 15-month follow-up. Analyses of the 15-month follow-up will be exploratory.

If we think we can recruit 300 participants, we will go for 1:2 (intervention: control) allocation. If we expect to get closer to 200 total subjects only, we will go for 1:1 allocation, because the number in the active arm is important for journal reviewers and we would then not have 100 in the intervention group under 1:2 randomization.

## 3.3. Recruitment of participants

The company representatives and/or the principal investigator will visit study sites and meet with the employees, if possible in small groups, to explain the purpose and rough procedures of the study to increase interest for this program beforehand.

Every month CRC will send out invitation letters and questionnaires to 200 employees, of whom 50 will be candidates (K6>=9), of whom 50 will be interviewed with CIDI by Dr Sasaki and Ms Kitagawa, of whom 40 will be eligible, of whom 30 will give the final informed consent. These 30 will be randomized to active treatment (n=10) or to waiting list (n=20). Nine telephone counselors will then have to initiate telephone CBT with 1-2 clients every month.

We will repeat this process for 9 months or until we recruit 100 clients in the telephone CBT arm.

The long wait-list and unequal allocation is defensible and explained by the limited number of competent telephone counselors available. The 9 counselors can start treating only 1-2 clients every month, i.e. 3-4 clients simultaneously at any given time (because one course of telephone CBT lasts 2-3 months); in order to recruit 100 clients, this means that 9 months of recruitment will be necessary for the 9 counselors. Those on the waiting list can receive the telephone CBT only after 15 months.

### 3.3.1. Assessment of eligibility

#### 1^st^ screening and preliminary IC

We will send out the invitation letter, written consent form, and the screening questionnaires (JCQ, HPQ, K6 and face sheet asking for the name, date of birth, sex, email address/telephone number, job category, current treatment for physical condition, current treatment for mental condition, whether he/she is currently on sick leave for any reason, and whether he/she would like to receive the feedback on the questionnaire results, candidate appointment times for CIDI) to all the workers of Office X and Factory Y of the company.

Those who consent to the screening questionnaire and interview will return the signed consent form and the screening questionnaires individually.

#### 2^nd^ screening

When the questionnaire is returned and the person scores 9 or more on K6, a CIDI interviewer will call him/her up and administer the CIDI. The CIDI will cover the mood disorder and substance abuse sections.

We will return the feedback based on CIDI, JCQ, HPQ and K6 to those who have asked for it.

#### Final IC

Those who scored 9 or more on K6 but did not meet diagnostic criteria for current major depression, for substance dependence in the past 12 months or for lifetime bipolar disorder will be contacted by the CRC by mail (and/or by telephone) and will be asked for further consent to participate in the intervention trial.

Those who express consent on the telephone will be sent by mail the IC explanation and consent form and the BDI2.

On the other hand, those who were found to be currently in major depressive episode will be excluded from this trial but will be contacted by the CRC, so that they would be advised to see an industrial physician or an extra-mural specialist. The CRC would explain by letter that, according to all the data accumulated so far, the subject was judged to profit more by seeing a specialist in person than by this telephone CBT.

### 3.3.2. Baseline assessments

In order for the baseline assessment to reflect the true baseline at start of intervention, we will use two prompts for the 1^st^ IC & screening questionnaire and the 2^nd^ IC and BDI2, each within 2 weeks. Those who do not respond by 3 weeks will be considered non-consenting.

The baseline assessments will therefore consist of the following variables.

#### From the questionnaires for the1st screening

1. Job Content Questionnaire 🡪 Job control, Job demand
2. Health and Productivity Questionnaire 🡪 Total hours worked for the past 4 weeks, Relative absenteeism for the past 4 weeks (% of the working hours absent), Relative presenteeism for the past 4 weeks (Effective hours worked)
3. K6
4. Date of birth
5. Sex
6. Job category
7. Current treatment for physical and/or mental conditions
8. Any sick leave in the past month
9. Expected to be on pregnancy leave, maternity leave, or nursing leave within 2 years

#### From the CIDI interview and the subsequent questionnaire:

CIDI results for mood and substance use disorders

BDI2 for the past 2 weeks

### 3.3.3. Informed consent

Written informed consent will be obtained from all participants included in this study after full disclosure and explanation of the purpose and procedures of the study. Candidates will be informed that their participation is totally voluntary, that even after voluntarily participating they can withdraw from the intervention or from both the intervention and the assessment at any time without stating the reason, and that neither participation nor withdrawal will lead to any advantage or disadvantage at the company.

Data of each participant will be handled with sequentially allocated numbers to keep the participant’s confidentiality and kept in a computer not connected to the Internet. The list of participants and their numbers will not be electronically stored but will be kept on a separate sheet stored in a safe, locked place and will be accessible to the CRC only.

The explanation for IC will emphasize the client’s susceptibility/vulnerability based on the screening data and potential benefit from the program, in order to enhance his/her adherence.

However, at the same time, we cannot oversell the program because we have the TAU condition as well.

A brochure will help. See pages 208 and 212 of Friedman, and examples from Geddes. Should be simple and in color. Avoid using the intervention name: otherwise, the participants in the control group will be demoralized. Asking for IC for questionnaire and IC for telephone CBT will, hopefully, increase patient adherence.

### 3.3.4. Intervention allocation

We will randomize eligible subjects to intervention and control groups at a ratio of 1:2. Randomization will be stratified on three variables, namely depression severity at baseline (BDI2=<19 vs >=20), absolute presenteeism prior to baseline (6 or higher vs 5 or lower, based on the obtained median of the eligible subjects in the pilot run) and center (Office X, Factory Y, Factory Z). Assuming that subjects are recruited from 3 sites, there will be a total of 12 strata. The study statistician will prepare 12 lists of random assignments ahead of time, one for each of the 12 strata. Each list will be blocked to ensure that the ratio of intervention to control is exactly 1:2 at set intervals within each stratum. The statistician will send these lists to the research assistant who is not involved in any other aspects of the study and who will build a spreadsheet that enables concealed sequential allocation and will be responsible for keeping these lists in the safe place.

Before randomizing an individual, the CRC will make a final determination of whether or not the person is eligible to be in the randomized trial. For example, prior to randomization, we will exclude those who score too low (BDI2=<9), which may well occur because of the difference between K6 and BDI2 and due to time elapsed for screening, because those who score too low at baseline will have less room for improvement. Once the CRC determines the eligibility of the participant, she will enter his/her registration number and the stratification variables to the spreadsheet which will automatically generate the allocation according to the pre-determined random sequence. The entered variables cannot be undone in order to guarantee allocation concealment.

Those randomized to control group will be put on a waiting list and after 15 months can receive the telephone CBT if they wish to do so.

One stratification factor is BDI2>=20 and BDI2<=19. This cutoff was chosen because our previous study suggests the score ranges 14-19 to be mildly ill, 20-28 to be moderately ill and 29- to be severely ill (Hiroe et al., 2005) and because our pilot study suggested that this is about the median BDI2 value for employees with K6>=9.

Another stratification factor is presenteeism, because this will be one of our primary outcomes and it is important to make sure that some effect can be seen, when in fact there is, probably among the more disturbed people.

# 4. INTERVENTIONS

## 4.1. Description and schedule

In order for the randomization and start of intervention to be as close as possible, CRC will endeavor to randomize the client the day after the 2^nd^ IC is returned, arrange the 1^st^ appointment and send out the appropriate manuals on the same day.

The telephone counseling makes use of a client manual and a client workbook. We have also prepared a counselor manual. All of these have been modeled after “Creating a Balance: A Step-by-Step Approach to Managing Stress and Lifting Your Mood” (Simon et al., 2006) which has been trialed in two large RCTs and found effective in alleviating major depression when combined with pharmacotherapy in the primary care (Simon et al., 2004) and in the workplace (Wang et al., 2007).

The telephone CBT consists of 8 sessions; 1 for introduction, 3 for BA, 3 for CR and 1 for summary. The contents of the program have been modified and adapted, based on the expert opinions of the PIs of this study and on the feedbacks received from clients in the pilot runs in order to make them more suitable for subthreshold depression among workers in the Japanese culture. For each session, the client has to make a preparation, receive a telephone counseling session for approximately 30-45 minutes, and subsequently do some homework.

The EAP program is the one which has already been in use by this company since 1997. The program includes a web site for mental and physical health, a p.r.n. telephone or in-person counseling service accessible to all the employees and on-site lectures on mental and physical health for managers and workers. For the year 2007, the average service provided per employee was 0.09 mental health self-check-ups on the web, 0.003 telephone counseling session, and 0.001 in-person counseling session. The company paid $3 per person per annum for this much service. In addition, Office X and Factory Y have part-time psychologists who administered 0.13 session per employee in 2006. We will not tell the providers of the EAP program about this telephone CBT trial, but at the same time we will not ask the participants to refrain from telling the EAP personnel about the trial if and when they do contact them.

## 4.2. Quality control of treatments

Maintaining quality of the intervention is very important to maximize the power of the study.

The three supervisors are qualified in cognitive therapy.

The three supervisors and the 9 telephone counselors have received two-day workshop with Evette Ludman, who developed the original telephone CBT program used in this project or another workshop modeled after the original one.

The psychologists (MSc, PhD and post-PhD levels) have had one individually supervised and two group-supervised cases before acting as counselors in the trial. The supervisors and the counselors will have regular conferences every two months.

All the sessions will be audiotaped. The adherence of the session contents will be checked by the counselor self-checklist, which will further be checked by the CRC on an on-going basis. A random 10% subset of the audiotaped sessions, from early/middle/late phases of the study, will be assessed by the supervisors using the same checklist, and reported back and discussed at the regular counselors’ meetings. The degree of adherence by an independent rater and the degree of concordance between the self-check and an independent assessor’s rating will be reported.

## 4.3. Measures of client adherence

The CRC will check for client adherence and notify the therapist and his/her supervisor if any of the following is noticed:

1. More than 3 weeks have elapsed since the allocation
2. More than 3 weeks have elapsed between sessions
3. K6 >=10 for any of the sessions at session #2 or afterwards
4. K6 >=6 for any of the sessions at session #5 or afterwards

# 5. ASSESSMENTS

## 5.1. Measures

### Beck Depression Inventory-II (BDI2)

The Beck Depression Inventory, originally published in 1961 (Beck et al., 1961), has been the most widely used self-report measure of depression severity. With the advent of the DSM-IV, the time frame and question items have been updated as the 2^nd^ edition of the BDI, and its reliability and validity have been confirmed (Beck et al., 1996). The reliability and validity of the Japanese version have been found to be excellent (Hiroe et al., 2005).

We will administer BDI2 at baseline, at 4-month follow-up (end of acute phase treatment) and at 15-month follow-up.

### Health and Productivity Questionnaire (HPQ)

The World Health Organization health and Productivity Questionnaire (HPQ) is a self-report instrument designed to estimate the workplace costs of health problems in terms of self-reported sickness absence (absenteeism) and reduced job performance (presenteeism). Validation studies have found documented significant associations (r=0.61 to 0.87) of HPQ work hours assessments with payroll records (Kessler et al., 2003a) and job performance assessments with supervisor ratings (r=0.52) (Kessler et al., 2004) and other administrative records (area under the curve, 0.58 to 0.72) (Kessler and Ustun, 2004).

We will administer HPQ at screening, at 4-month follow-up (end of acute phase treatment) and at 15-month follow-up.

### K6

K6 is a recently developed very short (6-item) self-report questionnaire to screen for common mental disorders (Kessler et al., 2002). It is based on modern item response theory methods and consists of questions that are maximally discriminative of respondents in the 90^th^-99^th^ percentile range of the general population distribution because it is known that between 5-10% of the population suffer from mental disorders at any point in time. Only items displaying constant psychometric characteristics across sociodemographic variation are included in the final model. K6 has been found to work as well as and better than some widely used screening questionnaires (Furukawa et al., 2003; Kessler et al., 2003b). The Japanese version has been validated (Furukawa et al., in press).

We will use K6 as the initial screening instrument. In addition, we will use K6 as a process measure in the course of the telephone CBT and administer it at the beginning of every session. Together with the screening, 4-month follow-up and 15-month follow-up data (which will all be administered to the control group as well), K6 will be one of the secondary outcome measures.

### Composite International Diagnostic Interview (CIDI)

The CIDI is a widely used fully-structured diagnostic interview for assessing mental disorders, to be used with the general population by trained lay interviewers (Robins et al., 1988). It has been successively updated to accommodate the DSM-IV. We used the most recent computerized version (Kessler and Ustun, 2004) and administered the sections for mood disorders and alcohol use. The concordance between the CIDI and standardized clinical assessments has been reported (Haro et al., 2006). The Japanese version of the HPQ and CIDI has been used in the World Mental Health Survey in Japan (Kawakami et al., 2005). [Norito, could you supplement? Is there any validation study?]

## 5.2. Follow-up visit description and schedule

### 5.2.1. Drop out from the intervention

The subject is free to withdraw from the intervention and/or the study at any time. However, we plan to make sure that the therapists and the CRC know how important it is to ask those who drop out of the intervention to complete the 4-month follow-up. Even if someone discontinues participation in the intervention, we will therefore make every effort to collect outcome data from them at the follow-ups at month 4 and at month 15.

If a new mental condition arises or the existing mental condition worsens, all the participants, whether during the telephone CBT or outside of the CBT, are free to utilize the EAP routinely made available by the company and/or seek professional help outside the company. In addition, they can call up the free hotline connected to the study center psychologist. If a new mental condition or worsening of the existing subthreshold depression occurs during the telephone CBT such that treatment outside the current program is advisable, the counselor must advise the subject to seek professional in-person help from the industrial physician, his/her home doctor or a mental health specialist. The participation in the intervention itself can be continued if the counselor and the subject see no conflict between the telephone CBT and the newly offered treatment, or discontinued should there be any perceived conflict or wish to withdraw expressed from the part of the subject. Even if the telephone CBT itself is discontinued, the subject is encouraged to take part in the 4- and 15-months follow-ups.

### 5.2.2. Drop out from the study

Those who withdraw consent to the follow-ups at 4 and/or 15 months will be considered dropouts from the study.

### 5.2.3. How to enhance adherence and reduce dropouts

Dropout from the intervention will be strictly distinguished and differentiated from dropout from the study. We will ask the client, so far as it is possible with him/her, that he/she respond to the final assessment even if he/she has chosen to stop the telephone counseling itself.

The explanation for IC will emphasize the client’s susceptibility/vulnerability based on the screening data and potential benefit from the program, in order to enhance his/her adherence.

A brochure (e.g. page 208, page 212 of the textbook) may help.

Taking IC twice will likely eliminate those half-hearted volunteers and/or also enhance adherence among the more determined.

We will send out New Year Cards to both the intervention and the control groups for the year 2010, which is right before the 15-month follow-up for the first cohort, and in 2011. We will send out a thank-you card after 4-month and 15-month follow-ups. It will be very good if we can give any reward, monetary or otherwise, especially to those people who respond to the 4-month [this is our primary outcome] and 15-month follow-up, but this needs to be negotiated with the sponsor.

## 5.3. Assessment of response variables

### 5.3.2. Independence of the self-reports

Our primary outcome, the BDI2 to be completed by the participant, is mailed back confidentially to the CRC and not to the therapist, in order to assure anonymity and independence of the ratings.

It is explicitly explained in the letter that these data will not be shared with the counselor, so honest disclosure is most welcome.

### 5.3.1. Data collection

#### 4-month follow-up (Acute-phase outcomes)

In the intervention group, the primary outcome will be measured at 4 months post randomization, i.e. approximately one month after the last session of the telephone counseling.

In the control group, the endpoint assessment will take place at 4 months post randomization.

The questionnaires will be mailed to the participants. If no response is received within 2 weeks, a second set of questionnaires with an appropriate cover letter will be mailed. If no response is still available, CRC will call the participants, both in the active treatment or control groups. Telephone response to the questionnaire battery is accepted.

#### 15-month follow-up

Both the intervention group and the control group will receive questionnaires at 15 months post randomization.

The CRC will first send out a notification postcard two weeks before actually sending out the questionnaires. Then the questionnaires will be mailed to the participants. If no response is received within 2 weeks, a second set of questionnaires with an appropriate cover letter will be mailed. If no response is still unavailable, CRC will call the participants, both in the active treatment or control groups.

### 5.3.3. Training of CIDI interviewers

CIDI will be administered by two interviewers with extensive prior training and experiences, and three interviewers who will have been trained and will have done a dozen pilot interviews.

All the other response variables are self-reports.

### 5.3.4. Quality control of assessments

Ongoing:

The CRC will check all the self-reports and get back to the participants should there be any missing data. The CRC will monitor the progress of all the therapy sessions and follow-ups according to the predefined criteria.

Periodic:

Cf. DSMC.

Post hoc:

All the CIDI interviews, both at baseline and at 15-month follow-up, will be audiotaped and a random 10% subset will be monitored for inter-rater reliability. The CIDI raters will not be blind to the treatment allocation for logistic reasons, because the CRC is responsible for approximately half of the CIDI interviews.

# 6. ANALYSES

## 6.1. Data analysis

### 6.1.1. Interim monitoring

No interim analysis will be performed, because we anticipate that the recruitment will be on-going over the course of the first 9 months and we will have recruited half of the planned sample size before we have any outcome data at all from the 4-month follow-up to review.

### 6.1.2. Preliminary Analyses

Our first set of analyses will check for out-of-range values and logical inconsistencies (someone reports on one question that he/she missed no days work in the prior month and then reports on a subsequent question that he/she missed 4 days in last month due to illness).

We will also compute summary statistics (mean, standard deviation, minimum, maximum, skewness) for each of our outcome variables and do a histogram plot to get a graphical picture of the distribution for each outcome. We will do this separately for outcomes at baseline, month 4, and month 15.

### 6.1.3. Analyses of Baseline Data

For continuous and ordinal variables, such as BD12 and HPQ, we will compute the means for the control and intervention groups and test whether they are significantly different using a t-test. For all yes/no baseline variables, we will compute the percentage yes in each of the two groups and compare using a 1 degree of freedom chi-square test. We may or may not report the p-values from these tests in our "Table 1", which will compare the groups at baseline. Whether or not we report them will depend on the journal that we submit the main outcome paper to since some journals want them reported and some do not.

### 6.1.4. Analyses of Primary Outcomes

Our two primary outcomes are the BD12 measure of depression severity and the HPQ measure of effective hours worked, both assessed at the month 4 follow-up. Both of these measures are continuous/ordinal variables so will compute the mean BD12 and mean HPQ at month 4 in the two groups, control and intervention.

We will first do unadjusted analyses to compare the means using a t-test. We will then do several adjusted analyses. First will use ANCOVA to compare the month 4 means on the outcome adjusting for baseline differences on the outcome. ANCOVA is simply another term for regression. For example, in one model, BD12 at month 4 will be the outcome and treatment group (a 0/1 variable) and baseline BD12 will the independent variables. These ANCOVA analyses provide estimates of "adjusted" month 4 group means as well as estimates of the adjusted difference in the group means (along with a p-value and confidence interval for the difference).

We will also do adjusted analyses which control for additional baseline variables, including variables which differ significantly between the control and intervention groups and variables which are strong predictors of the outcome under analysis. We will compare the estimated difference in group means and p-values to those obtained from the unadjusted analysis and the ANCOVA analyses which adjusts only for the baseline value of the outcome.

These analyses will be restricted to subjects who complete the month 4 follow-up (all of the statistical methods described above drop from the analysis those with a missing value on the outcome). Since a true intent-to-treat analysis estimates treatment effects based on data from all randomized subjects, not just those who complete a given follow-up, we will also do some mixed-model hierarchical linear models (HLM) to estimate treatment effects based on all subjects. NOTE: If the response rate is high at the month 4 follow-up (say at least 90%), bias due to missing data will likely be quite small and these analyses may not be necessary.

If it is necessary to do a HLM analysis, we will use what is called a mixed-model random coefficient model. Subjects are "random effects" with varying intercepts (baseline values) and vary slopes (rates of change from baseline to month 4). The dataset is structured so that each person has up to 2 records: one for baseline and one for follow-up. Even subjects who complete only the baseline enter into the analysis and are taken into account when estimating the month 4 mean in each group. For example, if subjects who do not complete the month 4 follow-up have higher baseline BD12 scores than subjects who do complete the month 4 follow-up, then the estimates of the month 4 means in this analysis will be higher than in the respondent only analyses described above. Such a model can be fit in SAS and other standard statistical packages such as STATA and SPSS.

For example, the random-coefficient ANCOVA model for BD12 would be:

BD12month4 = intercept + Beta1*treatment + Beta2 * BD12baseline

where the intercept and Beta2 parameters are modeled as random effects. Beta1 is a fixed effect and estimates the difference in the means in the intervention and control groups (i.e. estimates the treatment effect).

If conclusions based on the respondent-only and the intent-to-treat analyses are different, we will report intent-to-treat results.

### 6.1.5. Analyses of secondary outcomes

Analyses of continuous/ordinal secondary outcomes will be done in the same way as described above: first unadjusted comparison of groups means using t-tests, then ANCOVA comparisons of group means adjusting for baseline values of the outcome, and finally regression analyses which adjust for additional baseline variables. Depending on the response rates at month 4 (and whether these rates are similar in control and intervention groups), we may also compare group means using mixed-model HLM analyses.

For yes/no outcome variables, such whether or not a subject quit his/her job during follow-up, we will compare the percentages yes in the control and intervention group first using a chi-square test to do an unadjusted analysis. We will then use logistic regression to do an ANCOVA type analysis which adjusts only the baseline of the outcome. Lastly we will do adjusted logistic analyses which control for other baseline variables.

### 6.1.5. Analysis of non-response

We will do analyses to compare the baseline characteristics of subjects who complete the month 4 follow-up and subjects who do not complete the month 4 follow-up (completers and non-completers). We want to know if those who do not complete the month 4 follow-up differ in important ways from those who do. For example, it would be important to know if the non-completers have more severe depression at baseline. If so, non-completion would be more likely to bias the results, particularly if the completion rates differ between control and intervention.

For all of our variables in Table 1, we will compute means and percentages of the completers and non-completers for each treatment group at baseline. We will use t-tests to compare the means for continuous/ordinal variables in the completers and non-completers. For yes/no variables, we will compare the percentages in these two groups using a 1 d.f. chi-square test.

## 6.2. Termination policy

We are planning a recruitment phase of 9 months (extendable up to 12 months). We will then start the 1-year follow-up, to be implemented at 15 months after randomization for both the treatment and waiting list groups.

After this 15-month follow-up evaluation, those in the waiting list group who wish to receive telephone CBT can receive telephone CBT.

# 7. ORGANIZATION

## 7.1. Participating investigators

### 7.1.1. Project management team

#### Co-PI

Toshi A. Furukawa, MD, PhD (psychiatrist)

Masaru Horikoshi, PhD (clinical psychologist)

Norito Kawakami, MD, MPH (psychiatric epidemiologist)

#### Clinical research coordinator

Megumi Sasaki, PhD (clinical psychologist)

### 7.1.2. Data coordinating center

Dr Sasaki will be the only one at the Data coordinating center to be located within the Department of Psychiatry and Cognitive-Behavioral Medicine of Nagoya City University Graduate School of Medical Sciences.

### 7.1.3. Therapists and supervisors

#### Clinical supervisors

Dr Toshi A. Furukawa

Dr Masaru Horikoshi

Ayumi Horikoshi

#### CIDI interviewers

Ms Kitagawa, who has already been trained and has participated in a number of psychiatric epidemiological surveys

Dr Sasaki, who is also CRC, has been trained in CIDI

Mr Yuki Ooe has been trained in CIDI.

#### Telephone counselors

9 psychologists at MSc, PhD or post-PhD level (6 are certified clinical psychologists)

### 6.1.4. Statistical unit

Dr Toshi A. Furukawa will be responsible for the statistical analyses.

Mr Lou Grothaus will consult with Dr. Furukawa regarding the design and conduct of the study, what statistical analyses should be done and how to interpret the results, and the reporting of the results for publication.

## 8.2. Study administration

### 8.2.1. Steering committee

The CRC will summarize the progress of recruitment and data collection every month, and send out the abbreviated report to all the study collaborators. The project management team will meet every two months to monitor the progress of the study.

The progress of the study will also be reported to the counselors at their bimonthly conference in order to motivate them.

### 8.2.2. Data and Safety Monitoring Board (DSMB)

The DSMB will consist of the CRC, Mr. Lou Grothaus (statistical consultant), the two company representatives and a scientific colleague of Professor Furukawa who is familiar with clinical trials and is not involved in this study (person to be determined).

The DSMB will meet every 6 months after the first client is randomized. The purpose of the meeting will be to review the report prepared by the CRC.

CRC will prepare for the DSMB reports which monitor recruitment progress and data collection (e.g. % completing different follow-ups) and adverse events and their resolution..

We will prepare a free phone number for participants to call up, should they have any suicidal ideation and other significant worsening of their depression. (Cf. 2.4. Adverse events)

### 8.2.3. Funding organization

Sekisui Chemicals, Co.

# REFERENCES

Allart-van Dam, E., Hosman, C.M., Hoogduin, C.A., Schaap, C.P., 2007. Prevention of depression in subclinically depressed adults: follow-up effects on the 'Coping with Depression' course. J. Affect. Disord. 97, 219-228.

Beck, A.T., Steer, R.A., Brown, G.K., 1996. BDI-II: Beck Depression Inventory, Second Edition, Manual. The Psychological Corporation, San Antonia.

Beck, A.T., Ward, C.H., Mendelson, M., Mock, J., Erbaugh, J., 1961. An inventory for measuring depression. Arch. Gen. Psychiatry 4, 561-571.

Broadhead, W.E., Blazer, D.G., George, L.K., Tse, C.K., 1990. Depression, disability days, and days lost from work in a prospective epidemiologic survey. JAMA 264, 2524-2528.

Clarke, G.N., Hawkins, W., Murphy, M., Sheeber, L.B., Lewinsohn, P.M., Seeley, J.R., 1995. Targeted prevention of unipolar depressive disorder in an at-risk sample of high school adolescents: a randomized trial of a group cognitive intervention. J. Am. Acad. Child Adolesc. Psychiatry 34, 312-321.

Clarke, G.N., Hornbrook, M., Lynch, F., Polen, M., Gale, J., Beardslee, W., O'Connor, E., Seeley, J., 2001. A randomized trial of a group cognitive intervention for preventing depression in adolescent offspring of depressed parents. Arch. Gen. Psychiatry 58, 1127-1134.

Cuijpers, P., Smit, F., van Straten, A., 2007. Psychological treatments of subthreshold depression: a meta-analytic review. Acta Psychiatr. Scand. 115, 434-441.

Dimidjian, S., Hollon, S.D., Dobson, K.S., Schmaling, K.B., Kohlenberg, R.J., Addis, M.E., Gallop, R., McGlinchey, J.B., Markley, D.K., Gollan, J.K., Atkins, D.C., Dunner, D.L., Jacobson, N.S., 2006. Randomized trial of behavioral activation, cognitive therapy, and antidepressant medication in the acute treatment of adults with major depression. J. Consult. Clin. Psychol. 74, 658-670.

Druss, B.G., Marcus, S.C., Olfson, M., Tanielian, T., Elinson, L., Pincus, H.A., 2001. Comparing the national economic burden of five chronic conditions. Health Aff. (Millwood). 20, 233-241.

Furukawa, T.A., Kawakami, N., Saitoh, M., Ono, Y., Nakane, Y., Nakamura, Y., Tachimori, H., Iwata, N., Uda, H., Nakane, H., Watanabe, M., Naganuma, Y., Hata, Y., Kobayashi, M., Miyake, Y., in press. The performance of the Japanese version of the K6 and K10 in the World Mental Health Survey Japan. International Journal of Methods in Psychiatric Research.

Furukawa, T.A., Kessler, R.C., Slade, T., Andrews, G., 2003. The performance of the K6 and K10 screening scales for psychological distress in the Australian National Survey of Mental Health and Well-Being. Psychol Med 33, 357-362.

Geddes, J.R., Carney, S.M., Davies, C., Furukawa, T.A., Kupfer, D.J., Frank, E., Goodwin, G.M., 2003. Relapse prevention with antidepressant drug treatment in depressive disorders: a systematic review. Lancet 361, 653-661.

Greenberg, P.E., Kessler, R.C., Birnbaum, H.G., Leong, S.A., Lowe, S.W., Berglund, P.A., Corey-Lisle, P.K., 2003. The economic burden of depression in the United States: how did it change between 1990 and 2000? J. Clin. Psychiatry 64, 1465-1475.

Haro, J.M., Arbabzadeh-Bouchez, S., Brugha, T.S., de Girolamo, G., Guyer, M.E., Jin, R., Lepine, J.P., Mazzi, F., Reneses, B., Vilagut, G., Sampson, N.A., Kessler, R.C., 2006. Concordance of the Composite International Diagnostic Interview Version 3.0 (CIDI 3.0) with standardized clinical assessments in the WHO World Mental Health surveys. Int J Methods Psychiatr Res 15, 167-180.

Hiroe, T., Kojima, M., Yamamoto, I., Nojima, S., Kinoshita, Y., Hashimoto, N., Watanabe, N., Maeda, T., Furukawa, T.A., 2005. Gradations of clinical severity and sensitivity to change assessed with the Beck Depression Inventory-II in Japanese patients with depression. Psychiatry Res. 135, 229-235.

Judd, L.L., Akiskal, H.S., Paulus, M.P., 1997. The role and clinical significance of subsyndromal depressive symptoms (SSD) in unipolar major depressive disorder. J. Affect. Disord. 45, 5-17; discussion 17-18.

Judd, L.L., Schettler, P.J., Akiskal, H.S., 2002. The prevalence, clinical relevance, and public health significance of subthreshold depressions. Psychiatr. Clin. North Am. 25, 685-698.

Kawakami, N., Takeshima, T., Ono, Y., Uda, H., Hata, Y., Nakane, Y., Nakane, H., Iwata, N., Furukawa, T.A., Kikkawa, T., 2005. Twelve-month prevalence, severity, and treatment of common mental disorders in communities in Japan: preliminary finding from the World Mental Health Japan Survey 2002-2003. Psychiatry Clin. Neurosci. 59, 441-452.

Kessler, R.C., Akiskal, H.S., Ames, M., Birnbaum, H., Greenberg, P., Hirschfeld, R.M., Jin, R., Merikangas, K.R., Simon, G.E., Wang, P.S., 2006. Prevalence and effects of mood disorders on work performance in a nationally representative sample of U.S. workers. Am. J. Psychiatry 163, 1561-1568.

Kessler, R.C., Ames, M., Hymel, P.A., Loeppke, R., McKenas, D.K., Richling, D.E., Stang, P.E., Ustun, T.B., 2004. Using the World Health Organization Health and Work Performance Questionnaire (HPQ) to evaluate the indirect workplace costs of illness. J. Occup. Environ. Med. 46, S23-37.

Kessler, R.C., Andrews, G., Colpe, L.J., Hiripi, E., Mroczek, D.K., Normand, S.L., Walters, E.E., Zaslavsky, A.M., 2002. Short screening scales to monitor population prevalences and trends in non-specific psychological distress. Psychol Med 32, 959-976.

Kessler, R.C., Barber, C., Beck, A., Berglund, P., Cleary, P.D., McKenas, D., Pronk, N., Simon, G., Stang, P., Ustun, T.B., Wang, P., 2003a. The World Health Organization Health and Work Performance Questionnaire (HPQ). J. Occup. Environ. Med. 45, 156-174.

Kessler, R.C., Barker, P.R., Colpe, L.J., Epstein, J.F., Gfroerer, J.C., Hiripi, E., Howes, M.J., Normand, S.L., Manderscheid, R.W., Walters, E.E., Zaslavsky, A.M., 2003b. Screening for serious mental illness in the general population. Arch. Gen. Psychiatry 60, 184-189.

Kessler, R.C., Ustun, T.B., 2004. The World Mental Health (WMH) Survey Initiative Version of the World Health Organization (WHO) Composite International Diagnostic Interview (CIDI). Int J Methods Psychiatr Res 13, 93-121.

Paykel, E.S., Freeling, P., Hollyman, J.A., 1988. Are tricyclic antidepressants useful for mild depression? A placebo controlled trial. Pharmacopsychiatry 21, 15-18.

Richardson, K.M., Rothstein, H.R., 2008. Effects of occupational stress management intervention programs: A meta-analysis. J. Occup. Health Psychol. 13, 69-93.

Robins, L.N., Wing, J., Wittchen, H.U., Helzer, J.E., Babor, T.F., Burke, J., Farmer, A., Jablenski, A., Pickens, R., Regier, D.A., et al., 1988. The Composite International Diagnostic Interview. An epidemiologic Instrument suitable for use in conjunction with different diagnostic systems and in different cultures. Arch. Gen. Psychiatry 45, 1069-1077.

Rost, K., Smith, J.L., Dickinson, M., 2004. The effect of improving primary care depression management on employee absenteeism and productivity. A randomized trial. Med. Care 42, 1202-1210.

Schoenbaum, M., Kelleher, K., Lave, J.R., Green, S., Keyser, D., Pincus, H., 2004. Exploratory evidence on the market for effective depression care in Pittsburgh. Psychiatr. Serv. 55, 392-395.

Simon, G.E., Ludman, E.J., Tutty, S., 2006. Creating a Balance: A Step-by-Step Approach to Managing Stress and Lifting Your Mood. Trafford Publishing, Victoria, British Columbia, Canada.

Simon, G.E., Ludman, E.J., Tutty, S., Operskalski, B., Von Korff, M., 2004. Telephone psychotherapy and telephone care management for primary care patients starting antidepressant treatment: a randomized controlled trial. JAMA 292, 935-942.

Spek, V., Nyklicek, I., Smits, N., Cuijpers, P., Riper, H., Keyzer, J., Pop, V., 2007. Internet-based cognitive behavioural therapy for subthreshold depression in people over 50 years old: a randomized controlled clinical trial. Psychol Med 37, 1797-1806.

Stewart, W.F., Ricci, J.A., Chee, E., Hahn, S.R., Morganstein, D., 2003. Cost of lost productive work time among US workers with depression. JAMA 289, 3135-3144.

van der Klink, J.J., Blonk, R.W., Schene, A.H., van Dijk, F.J., 2001. The benefits of interventions for work-related stress. Am. J. Public Health 91, 270-276.

Wang, P.S., Beck, A., Berglund, P., Leutzinger, J.A., Pronk, N., Richling, D., Schenk, T.W., Simon, G., Stang, P., Ustun, T.B., Kessler, R.C., 2003. Chronic medical conditions and work performance in the health and work performance questionnaire calibration surveys. J. Occup. Environ. Med. 45, 1303-1311.

Wang, P.S., Simon, G.E., Avorn, J., Azocar, F., Ludman, E.J., McCulloch, J., Petukhova, M.Z., Kessler, R.C., 2007. Telephone screening, outreach, and care management for depressed workers and impact on clinical and work productivity outcomes: a randomized controlled trial. JAMA 298, 1401-1411.

Wells, K.B., Stewart, A., Hays, R.D., Burnam, M.A., Rogers, W., Daniels, M., Berry, S., Greenfield, S., Ware, J., 1989. The functioning and well-being of depressed patients. Results from the Medical Outcomes Study. JAMA 262, 914-919.

Willemse, G.R., Smit, F., Cuijpers, P., Tiemens, B.G., 2004. Minimal-contact psychotherapy for sub-threshold depression in primary care. Randomised trial. Br. J. Psychiatry 185, 416-421.

World Health Organization, 2004. World Health Report 2004.
